# The effect of S427F mutation on RXRα activity depends on its dimeric partner†

**DOI:** 10.1039/d1sc04465f

**Published:** 2021-10-08

**Authors:** Ioannis Galdadas, Vangelis Bonis, Paraskevi Vgenopoulou, Michail Papadourakis, Panos Kakoulidis, Georgia Stergiou, Zoe Cournia, Apostolos Klinakis

**Affiliations:** a Biomedical Research Foundation Academy of Athens Athens Greece aklinakis@bioacademy.gr zcournia@bioacademy.gr; b Data Science and Information Technologies, Department of Informatics and Telecommunication, National and Kapodistrian University of Athens Athens Greece

## Abstract

RXRs are nuclear receptors acting as transcription regulators that control key cellular processes in all tissues. All type II nuclear receptors require RXRs for transcriptional activity by forming heterodimeric complexes. Recent whole-exome sequencing studies have identified the RXRα S427F hotspot mutation in 5% of the bladder cancer patients, which is always located at the interface of RXRα with its obligatory dimerization partners. Here, we show that mutation of S427 deregulates transcriptional activity of RXRα dimers, albeit with diverse allosteric mechanisms of action depending on its dimeric partner. S427F acts by allosteric mechanisms, which range from inducing the collapse of the binding pocket to allosteric stabilization of active co-activator competent RXRα states. Unexpectedly, RXR S427F heterodimerization leads to either loss- or gain-of-function complexes, in both cases likely compromising its tumor suppressor activity. This is the first report of a cancer-associated single amino acid substitution that affects the function of the mutant protein variably depending on its dimerization partner.

## Abstract 2

A cancer-associated missense mutation in the nuclear receptor RXRα acts by allosteric mechanisms and impacts differently the activity of its dimers, depending on the dimerization partner.

## Graphical abstract

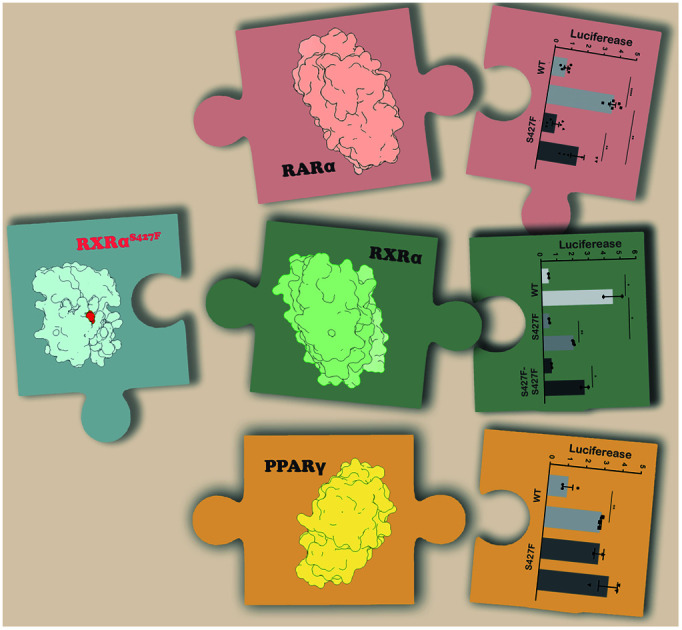

## Introduction

Bladder cancer is the second most common urogenital cancer with 550 000 new cases every year worldwide.^1^ Whole-exome sequencing of bladder cancer patients identified a hotspot mutation within the gene encoding the retinoid X receptor α (RXRα) involving Ser427 changing to Phe and less frequently to Tyr (RXRα^S427F/Y^).^2^

RXRα is a nuclear receptor of the vitamin A metabolite 9-cis retinoic acid (9-cis RA), and serves as an obligate homo- and hetero-dimerization partner for many subfamily 1 nuclear receptors, including the retinoic acid receptor α (RARα) and peroxisome proliferator-activated receptor γ (PPARγ).^3^ These protein dimers can then bind to target response elements consisting of a direct repetition of a half-site motif (5′-AGGTCA-3′) with an intervening spacer of 1–5 bp (DR1–5) leading to regulation of gene expression for various biological processes.^4^ Thus, RXRα, as well as RARα and PPARγ, function as ligand-activated transcriptional factors consisting of a ligand-binding domain (LBD) linked through a hinge region to a DNA binding domain (DBD). The LBD of these three receptors exhibits the canonical folding of nuclear receptors in which a single β-turn and 12 α-helices (H1–H12) enclose a hydrophobic binding pocket (Fig. S1†).

Ligands control allosterically the interactions of nuclear receptors with coactivators and corepressors by altering the conformation of the short helix H12, also referred to as AF2 – activation function 2, at the carboxy-terminal end of the LBD.^5^ In the absence of a ligand, H12 is in an open conformation that enables binding of co-repressors. Upon agonist binding, H12 undergoes a conformational change that leads to the formation of a novel surface to which coactivator proteins can dock through a short helix that contains the consensus LxxLL motif.^6^ Recent studies have indicated that RXRα^S427F^ allosterically activates the PPARγ H12 helix in the absence of 9-cis RA.^7,8^ This could explain the strong PPARγ transcriptional signature in bladder tumors bearing RXRα^S427F/Y^ mutations.^2^ However, it is yet unknown how the S427F mutation on RXRα exerts its biological actions on other heterodimers, such as the RXRα–RARα as well as the homodimer RXRα–RXRα.

Examination of several crystal structures that exist in the literature reveals that the S427F mutation lies at the interface between homo- and heterodimer RXRα complexes. Thus, the obvious hypothesis for the S427F mutant mechanism of action is the alteration of its dimerization capability. In order to assess the effect of the S427F mutation on the ability of RXRα to dimerize with its obligate partners, we used a combination of biochemical experiments and Molecular Dynamics (MD) simulations to further elucidate the effect of the S427F mutation on the structure and dynamics of RXRα in its homodimeric and heterodimeric form with RARα and PPARγ. Our results indicate a differential allosteric effect of the S427F mutation on the RXRα homo/heterodimers, which depends on the dimeric partner.

## Results

### Mutation of Ser 427 of RXRα does not affect its DNA binding activity

The crystal structures of the full-length nuclear receptor complexes of RARβ–RXRα and PPARγ–RXRα show that the DBD and LBD regions of RARβ and PPARγ are physically connected through a DBD-LBD linker. Therefore, we first asked whether the S427F mutation affects DNA binding. To investigate this, we expressed wild-type (WT) and mutant (S427F) RXRα in bacteria *Escherichia coli* tagged with glutathione *S*-transferase (GST) to facilitate protein purification. Purified recombinant proteins (RXRα^WT^ and RXRα^S427F^) were used in electrophoretic mobility shift assays (EMSA). In EMSA experiments, DNA binding proteins are co-incubated *in vitro* with a radiolabeled oligonucleotide containing cognate DNA binding sites, while the binding reaction is electrophoresed under non-denaturing conditions in a polyacrylamide gel. The protein-bound oligonucleotide migrates slower than the free one, generating a “gel shift” when exposed to an autoradiograph. For our experiments, as DNA target we used an oligonucleotide containing a DR1 site, which can be bound mainly by RXR homodimers (and RXR–PPARγ heterodimers). As Fig. S2† indicates, both RXRα^WT^ and RXRα^S427F^ bind equally well their cognate target sequence (lanes 1 and 6), indicating that the S427F mutation does not affect the DNA binding capacity of RXRα. The binding is specific, because it can be outcompeted by an excess (5× and 25×) of unlabeled DR1-containing oligonucleotides (lanes 2–3 and 7–8). Competition with oligonucleotides containing DR1 half-sites, which cannot be bound by RXR dimers, fail to compete for binding by RXRα^WT^ (lanes 4–5). However, we cannot exclude the possibility of RXRα^S427F^ binding as a monomer, as indicated by a moderate competition with unlabeled half sites (lanes 9–10). Mixtures of RXRα^WT^ and RXRα^S427F^ at different ratios do not affect binding efficiency, again supporting the conclusion that the DNA binding capacity is not affected by this point mutation.

After we confirmed that the S427F mutation does not affect DNA binding of RXRα, we focused on the LBDs of RXRα complexed with RARα or PPARγ and of the RXRα homodimer. Through extensive, unbiased MD simulations spanning the μs timescale for both the wild type and mutant RXRα in its homodimeric and heterodimeric form with RARα and PPARγ, we were able to determine mechanistic details of the effect of the S427F mutation on the activity of the different dimers and verify these with functional experiments.

### S427F substitution leads to less active RXRα–RARα complexes

**Fig. 1:**
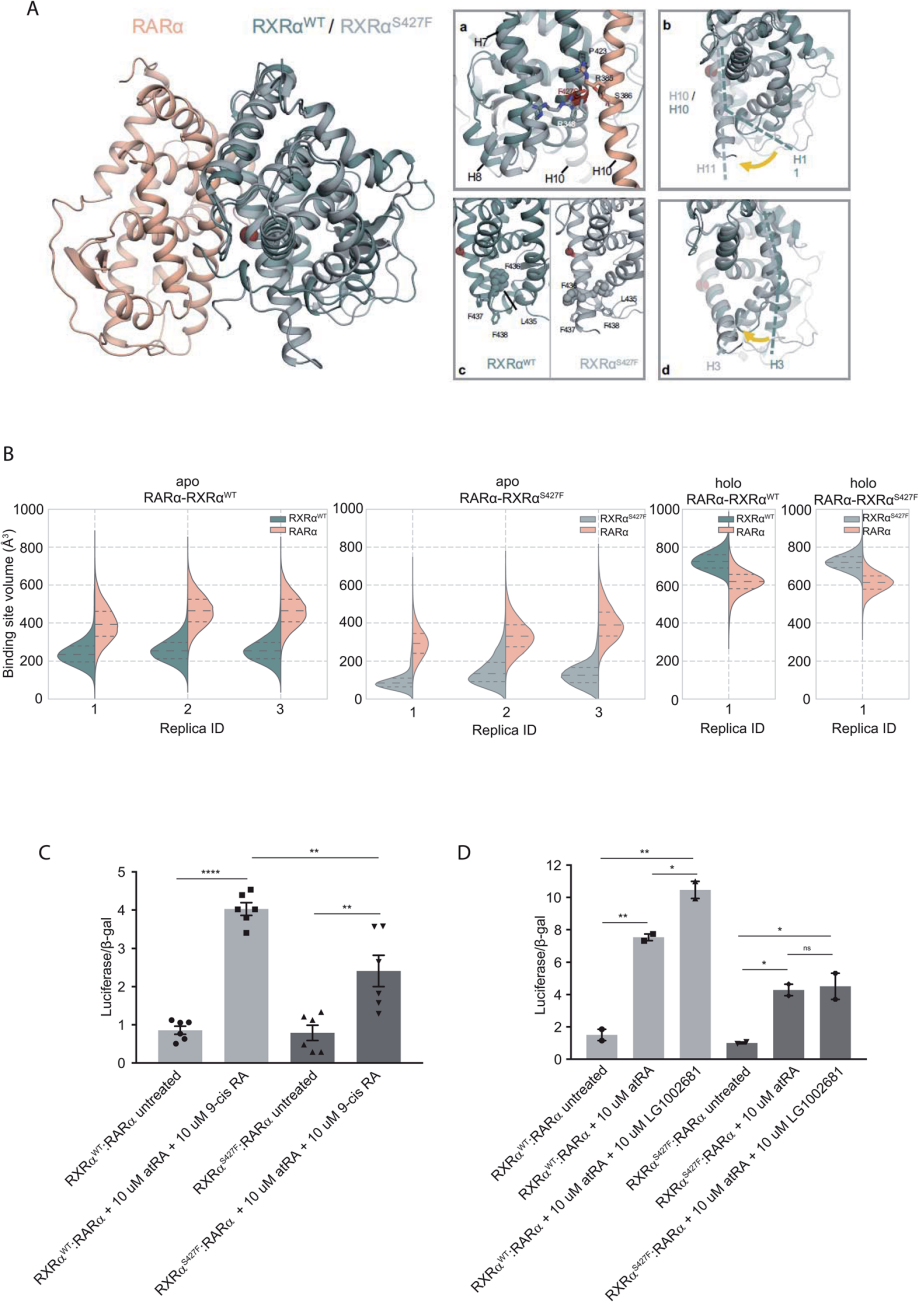
Computational and functional study of RXRα–RARα heterodimers. (A) Structural rearrangements on the LBD of RXRα–RARα heterodimer upon the RXRα S427F mutation. The position of the S427F mutation is depicted through a red sphere. (a) Interaction sites of Ser427 and Phe427, (b) Movement of RXRα H11, (c) rearrangement of the LFFF motif of H11, (d) movement of H3. (B) Violin plots showing the distribution of the volume of the binding-site of RXRα and RARα in the different replica simulations (Table S1†). The dashed line in the middle indicates the median of the distribution, while the other two dashed lines represent the interquartile ranges. Each RXRα^WT^ monomer is colored with green, each RXRα^S427F^ monomer is colored in grey and each RARα monomer in pink. (C) and (D) Boxplot indicating relative luciferase activity of the WT and mutant RXRα in heterodimers with RARα in response to 9-cis RA (C) and LG1002681 (D). In the *Y* axis is shown the normalized activity relatively to cells transfected with the DR5 reporter alone. * indicates *p* value < 0.05; ** indicates *p* value < 0.01; **** indicates *p* value < 0.0001. Student's *t*-test was used.

To assess the effect of the S427F mutation in the presence of an agonist, we ran the simulations in the absence (apo) and presence of 9-cis RA (holo) bound to the binding site of each monomer (Table S1†). For these simulations, we used the crystal structure of the RARα in its agonist conformation in complex with RXRα in an inactive conformation (resembling its homotetrameric state), where H12 points to the solvent (PDB ID 3A9E).^25^ Over the course of the apo RXRα^WT^–RARα simulations, the side chain of Ser427 engages predominantly in a hydrogen bond with the backbone of Pro423 (Fig. 1A(a)), which lies on the RXRα–RARα interface, while it also forms hydrogen bonds occasionally with Arg348 of H7 (Fig. S3†). The removal of the co-crystalized ligand leads to a gradual relaxation of the side chains of the binding-site residues in the void that the ligand leaves. After 300 ns, the conformation of the apo RXRα^WT^ converges towards its auto-inhibitory conformation, in which H12 binds to the co-factor binding-site resembling PDB ID:1DKF (Fig. S4†). Interestingly, Leu435 and Phe436 of H11 rotate with respect to the initial conformation in such a way that they pack themselves between H10 and H3, maintaining the relative position of the two helices (Fig. 1A(c)). This movement underlines the regulatory role of H11, which can be positioned differentially by distinct ligands or the absence of them thereby, controlling the position of H3 and the packing of H12 in the different conformers.

Because Ser427 is located at the dimer interface and outside the ligand-binding pocket, it has been suggested that mutations of this residue are unlikely to alter the ligand binding properties of RXRα.^8^ However, according to all our apo RXRα^S427F^–RARα simulations, H11 almost immediately rejoins with H10 (Fig. 1A(b)) allowing H3 to collapse into the hydrophobic ligand-binding site (Fig. 1A(d)). Specifically, the N-terminal part of H3 bends around H5, causing the binding site to be no longer ligand accessible. Then, H12 rotates gradually from the solvent back against the main body of the LBD and adopts a conformation similar to the one observed in the WT simulations. The closure of the binding site of RXRα due to the movement of H3 is reflected in the decrease of the binding site volume (Fig. 1B), which can prevent the RXRα ligand 9-cis RA from binding to the cognate pocket under physiological conditions. The inability of RXRα^S427F^ to bind 9-cis-RA was also confirmed using docking calculations (Table S2 and Fig. S5†) which showed that 9-cis RA demonstrated positive binding free energies when bound to RXRα^S427F^.

Proline residues usually cause kinks in α-helices, which leave the *i*+4 backbone carbonyl without its typical hydrogen bond donor and have been reported to play functional roles in proteins.^9,10^ In RXRα^WT^, the loss of the *i*+4 backbone hydrogen bond between Pro423 and Ser427 is compensated by a hydrogen bond with the side chain of Ser427 (Fig. S3†), which stabilizes the kinked H10. However, the loss of Ser427–Pro423 interaction upon mutation causes a subtle tilt in the curvature of H10 of around 3° (Fig. S6†), which triggers the rejoining of H11 with H10. Over the course of the RXRα^S427F^–RARα simulations, the side chain of Phe427 participates in a π-cation interaction with Arg348 of H7 (Fig. S3†), which increases the coupling of the motions of H10 and H7.

NMR resonances corresponding to residues within the apo-RXRα^WT^ ligand-binding site and H12 surface have indicated that these regions exist as a dynamic ensemble of conformations, and binding of 9-cis RA to RXRα stabilizes the ligand-binding pocket and H12 surface.^11^ Our holo RXRα/LG100754–RARα/atRA simulations are in line with this observation, as the overall structure of the receptor stays intact throughout the simulations. In the case of RXRα^WT^–RARα, where the antagonist LG100754 is bound to RXRα^WT^, H12 assumes an autoinhibitory position between H3 and H4 in the co-factor binding-site (Fig. S7†). In the case of RXRα^S427F^–RARα, Leu455 of H12 interacts with Leu276 and Val280 of H3 (Fig. S7†) and the position of H12 resembles an autoinhibitory position, even though it has not converged to the exact conformation during the simulation. Unlike the apo RXRα^S427F^–RARα, where residues H10 and H3 form direct interactions, in the holo state, the propoxy group of LG100754 extends between the two helices and maintains their relative position.

To explore the functional implications of the predictions that arose from the simulations, we employed a cell-based functional assay which relies on luciferase reporters with DR1 or DR5 elements controlling the firefly luciferase gene expression. The activity in response to combinations of RARα, RXRα and PPARγ can be quantitated in cell extracts in the form of luminescence (see below).

The RXRα–RARα heterodimer is considered nonpermissive,^12^ indicating that the RARα binding of its cognate ligand, all-*trans* retinoic acid (atRA), is required for dimer activity. Unlike other nonpermissive dimers however, subsequent 9-cis RA binding to RXRα boosts the activity of RXRα–RARα complex by 20–30%.^13^ If 9-cis RA cannot be bound by the RXRα in RXRα^S427F^–RARα heterodimers, then the expectation is that the transcriptional activity of the RXRα^S427F^–RARα heterodimeric complex in the presence of both ligands will be 20–30% lower than the RXRα^WT^–RARα one. Indeed, luciferase assays show that following concomitant treatment with atRA and 9-cis RA, the RXRα^S427F^–RARα heterodimeric complex leads to 40% lower luciferase activity (2.4× *vs.* 4×) in comparison to the RXRα^WT^–RARα complex (Fig. 1C). Because 9-cis RA can also bind RARα, we repeated the experiment using the synthetic rexinoid LG1002681, which binds exclusively to RXRs. Similar to 9-cis RA, LG1002681 also induced the activity of the RXRα^WT^–RARα complex by approximately 40% (7.5× *vs.* 10.5×). Contrary to this, LG1002681 failed to induce the transcriptional activity of the RXRα^S427F^–RARα complex beyond the activation achieved by atRA alone, strongly indicating that indeed, RXRα^S427F^ cannot bind its ligand, at least not as efficiently (Fig. 1D). It should be pointed out that in a recent study, the activity of RXRα^S427F^–RARα was also shown to be lower than that of RXRα^WT^–RARα heterodimer; however, the authors did not report it as a statistically significant finding.^7^

### S427F substitution leads to more stable but less active RXRα homodimers

Because RXRα mutations are not homozygous while RXRα can form homodimers, it is expected that all three combinations of mutant and WT homodimers will coexist in mutant cells. To address the effect of the S427F mutation in RXRα homodimers, in our MD simulations we used the crystal structure of the LBD of RXRα bound to the rexinoid agonist BMS649 (PDB ID 1MVC)^24^ and introduced the S427F mutation in one or both monomers.

It has been reported that the apo RXRα^WT^ is found predominately as a tetramer, which dissociates to its monomers upon addition of 9-cis RA.^14^ In a recent report, the RXRα^S427F^ was found predominately in its monomeric form.^8^ The absence of RXRα^S427F^ homodimers was rationalized based on the crystal structure of the RXRα^WT^, which indicates that the bulky phenylalanine would disrupt the RXRα^S427F^ homodimer interface. Our EMSA experiments (Fig. S2†), however, suggest that this is not the case. Not only do we observe binding, but also the existence of two clearly distinct shifts indicates the presence of two different DNA complexes (Fig. S2†). It has been previously shown that, in the absence of its ligand, RXRα binds DNA preferentially as a tetramer and then as a dimer.^15^ Thus, although we cannot exclude the possibility that RXRα^S427F^ deviates from this pattern, we speculate that the observed shifts can only correspond to RXRα homodimers and homotetramers.

**Fig. 2:**
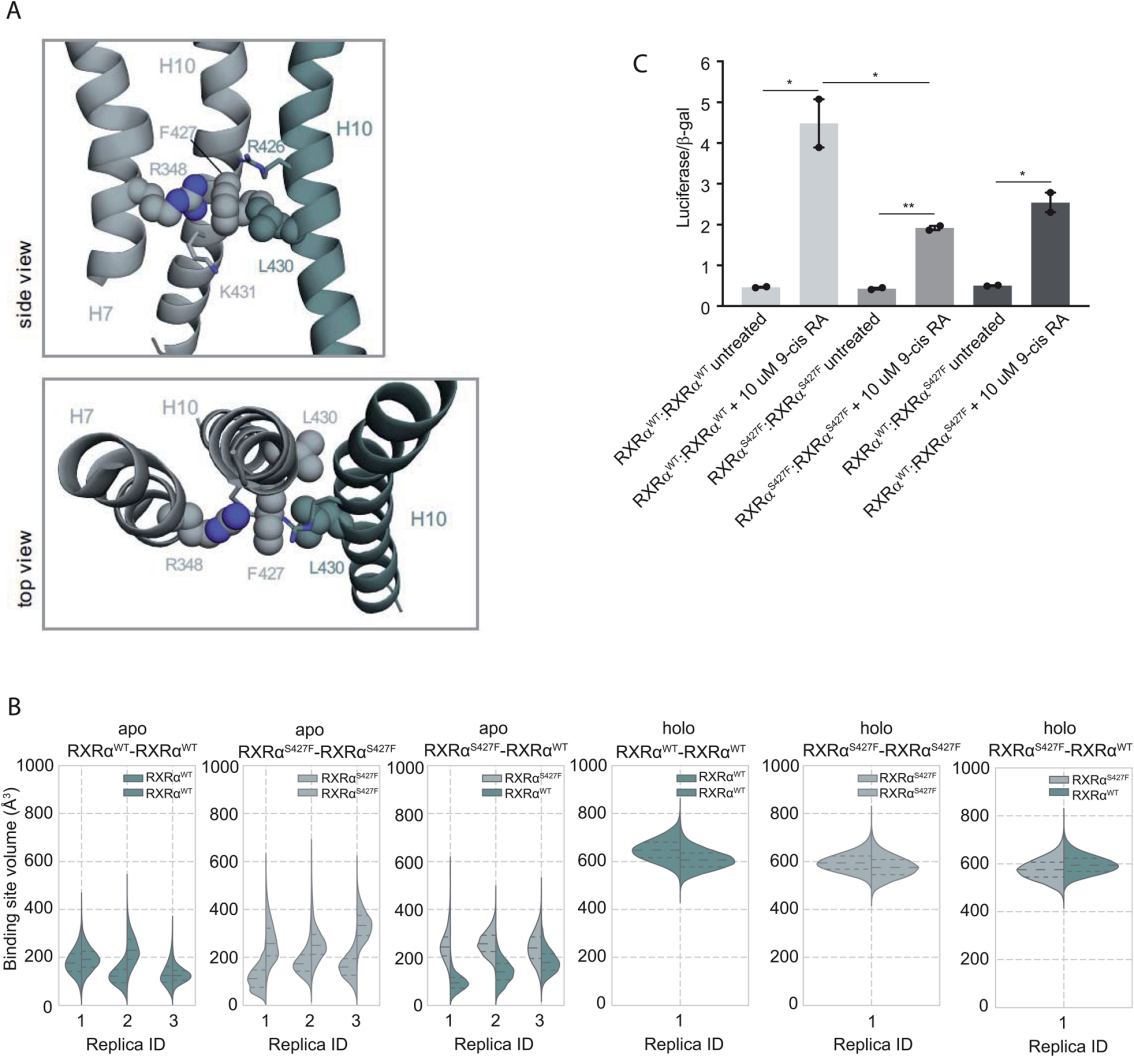
Computational and functional study of RXRα–RXRα homodimers. (A) Effect of the RXRα S427F on the LBD of RXRα–RXRα homodimer. Side and top view of the arrangement of Phe427 in the interface of RXRα^S427F^–RXRα^WT^ and RXRα^S427F^–RXRα^S427F^ over the course of the simulations. (B) Violin plots showing the distribution of the volume of the binding-site of RXRα^WT^ and RXRα^S427F^ in over the course of the simulations (Table S1†). The dashed line in the middle indicates the median of the distribution, while the other two dashed lines represent the interquartile ranges. Each RXRα^WT^ monomer is colored with green and each RXRα^S427F^ monomer is colored in grey. (C) Boxplot indicating relative luciferase activity of the WT and mutant RXRα homodimers in response to 9-cis RA. In the *Y* axis is shown the normalized activity relatively to cells transfected with the DR1 reporter alone. * indicates *p* value < 0.05; ** indicates *p* value < 0.01. Student's *t*-test was used.

Moreover, according to our simulations, Phe427 can be accommodated on the interface and also is in contact with Leu430 of the other monomer introducing a network of stabilizing hydrophobic and π-cation interactions between Phe427, Leu430, and Arg426 of the two monomers (Fig. 2 and S8†). Interestingly, Leu430 of RXRα is substituted with Ala in the case of RARα and Thr in the case of PPARγ, neither of which have a side chain long enough to interact effectively with Phe427. Similar to the case of RXRα–RARα, the side chain of S427 in RXRα^WT^–RXRα^WT^ and RXRα^S427F^–RXRα^WT^ forms a hydrogen bond with the backbone of Pro423 and the side chain of Arg426 (Fig. S8†). In fact, we observe that the interaction energy between the monomers of the RXRα^WT^–RXRα^S427F^ or RXRα^S427F^–RXRα^S427F^ dimers is stronger than that of the RXRα^WT^–RXRα^WT^ (Fig. S9†). The reported interaction energy between the monomers suggests that the mutation stabilizes the homodimer. Given that the mutation does not affect DNA binding efficiency (see above), we hypothesize that genomic RXR bindings sites will be more frequently occupied by the more stable RXRα^WT^–RXRα^S427F^ and RXRα^S427F^–RXRα^S427F^ dimers leading to reduced expression of the respective target gene.

Over the course of the simulations, we observe that the volume of the binding site in the RXRα^S427F^–RXRα^S427F^ and RXRα^WT^–RXRα^S427F^ dimers is affected (Fig. 2B), possibly impacting the binding of 9-cis RA in the pocket. The average volume of the binding pocket of each RXRα monomer for the three replica MD simulations of the RXRα–RXRα dimers (Table S3†) as well as the independent volume of each RXRα monomer in all replica MD simulations of the RXRα–RXRα dimers (Table S4†) can be found in the ESI.† Docking calculations (Table S5 and Fig. S10†) performed on the RXRα homodimers show that ligand binding demonstrates different behaviour depending on the presence of the S427F mutation. Docking scores in RXRα^WT^–RXRα^S427F^ showed that 9-cis RA can bind weakly to the RXRα^WT^ binding pocket (−3.76 kcal mol^−1^ on average for the three replicas, Table S5†), but cannot bind to the RXRα^S427F^ counterpart at all (predicted Δ*G* > 0 kcal mol^−1^). On the contrary, in the RXRα^WT^–RXRα^WT^ homodimer, 9-cis RA demonstrated negative docking scores for both monomers (−7.24 kcal mol^−1^ on average for the three replicas). Finally, 9-cis RA binds only slightly to the binding pocket of one of the two RXRα^S427F^–RXRα^S427F^ dimers (−1.88 kcal mol^−1^ on average, Table S5†), while it does not bind to the other dimeric partner (predicted Δ*G* > 0 kcal mol^−1^). In summary, in both RXRα^WT^–RXRα^S427F^ and RXRα^S427F^–RXRα^S427F^ dimers, only one of the monomers can bind 9-cis RA, due to binding pocket volume changes. This implies that both dimers are partially active at comparable levels.

This is corroborated by our luciferase assays. As Fig. 2C indicates, cells transfected with RXRα^WT^ and a DR1 luciferase reporter show an increase of 4.48-fold upon 9-cis RA treatment, while cells expressing the RXRα^S427F^ respond poorly (1.92-fold). Transfection with equimolar amounts of wt and mutant RXRα show an intermediate response to the ligand (2.54-fold). Based on the stoichiometry, in the wt and mutant co-transfection, the contribution of the wt : wt and mut : mut dimers represent each 25% of the observed luciferase activity, while the remaining 50% comes from the wt:mut heterodimer. Based on the activity in the wt and mut alone transfections, the wt : wt dimer would contribute 1.12 fold increase (25% of 4.48-fold), the mut : mut would add 0.48-fold (25% of 1.92), while the remaining 0.94-fold from the 2.54-fold in the wt : mut co-transfection would come from the 50% of wt–mut pair (1.88-fold increase for the 100%). This implies that the mut : mut and wt : mut dimers are equally active (1.92 *vs.* 1.88-fold). Collectively, the molecular simulations and the luciferase experiments indicate that RXRα^WT^–RXRα^S427F^ dimers show comparable activity with the RXRα^S427F^–RXRα^S427F^ ones.

The different binding activity of 9-cis RA between RXRα^WT^–RXRα^WT^, RXRα^S427F^–RXRα^S427F^ and RXRα^WT^–RXRα^S427F^ homodimers could also be explained from a network of residues that connects Trp305 of both monomers. Trp305 belongs to helix H5 of RXRα and it has been previously shown to mediate contacts with the bound ligand in the active RXR^WT^ homodimer. Moreover, in PPRAγ/RXRα heterodimers, helix Η5 of RXRα is an important part of the LBD core that transmits PPARγ allosteric signals from the dimer interface, that are induced from PPARγ agonists to the RXRα ligand-binding pocket. Ser427 of the RXRα^WT^–RXRα^WT^ homodimer was a part of the network of residues connecting the two tryptophans, while this was not the case for Ser427 of the RXRα^S427F^–RXRα^WT^ dimer or for Phe427 of the RXRα^S427F^–RXRα^S427F^ homodimer (Fig. S12†). Thus, the disruption of this network in RXRα^WT^–RXRα^S427F^ and RXRα^S427F^–RXRα^S427F^ could elucidate a possible allosteric mechanism that affects the binding of 9-cis RA in the RXRα pocket.

Finally, data from the apo MD simulations show that at least 1.5 μs are required for equilibrating the RXRα homodimers. RMSD plots as a function of time show that RXRα homodimers as a whole remain stable over the course of the MD simulations both in the presence and absence of 9-cis RA (Fig. S12†), however, the binding pockets of the apo RXRα^S427F^–RXRα^WT^ homodimer sample different conformations over the first 1.5 μs of the MD simulations (Fig. S13†), indicating local instability. Indeed, in a recent study by Yang *et al.*,^16^ it is shown that the apo-RXRα LBD homodimer displays a single thermal unfolding transition much lower than the average value of small globular proteins. On the other hand, when 9-cis RA is bound, RXRα homodimers retain more stable binding sites (Fig. S14†), also confirmed by differential scanning calorimetry and differential scanning fluorimetry experiments showing an increase in the free energy of the complex as a result of a more favorable entropic change due to interactions between the rexinoid and hydrophobic residues in the binding pocket.^16^ Finally, RXRα heterodimers with PPARγ and RARα are more stable, with respect to the homodimer, as indicated by interaction energy plots (Fig. S15†), where the homodimer interaction energy is significantly smaller. As a result, PPARγ and RARα might be RXRα preferential partners while waiting for the ligand.

### S427F substitution leads to constitutively active RXRα–PPARγ complexes

It has been demonstrated that the hotspot mutations S427F/Y in RXRα induce the activation of the PPRAγ/RXRα pathway in bladder cancer, leading to suppression of cytokine secretion from cancer cells.^8^ The crystal structure of the PPARγ/RXRα^S427F^ bound to 9-cis RA (PDB ID 5JI0)^1^ suggests that the mutation does not affect the organization of the residues around the site of the mutation, but the aromatic interaction between RXRα S427F and the terminal tyrosine Tyr477 found in all PPARs is responsible for the activation of PPARγ and stabilization of the heterodimer.^7,8^

During the first 80 ns of our RXRα^S427F^–PPARγ simulation, PPARγ Tyr477 forms a π–π interaction with Phe427 of RXRα^S427F^ (Fig. S16†), consistent with what has been observed in previous simulations of the same length.^7^ After that and until the end of the simulation, the side chain of PPARγ Tyr477 rotates and starts interacting with residues of H10 of PPARγ. This rotation brings Asp475 of PPARγ close to Arg348 of RXRα, which in turn forms a π-cation interaction with Phe427 (Fig. S16†). The interplay of these two states stabilizes the active conformation of H12 of PPARγ.

**Fig. 3:**
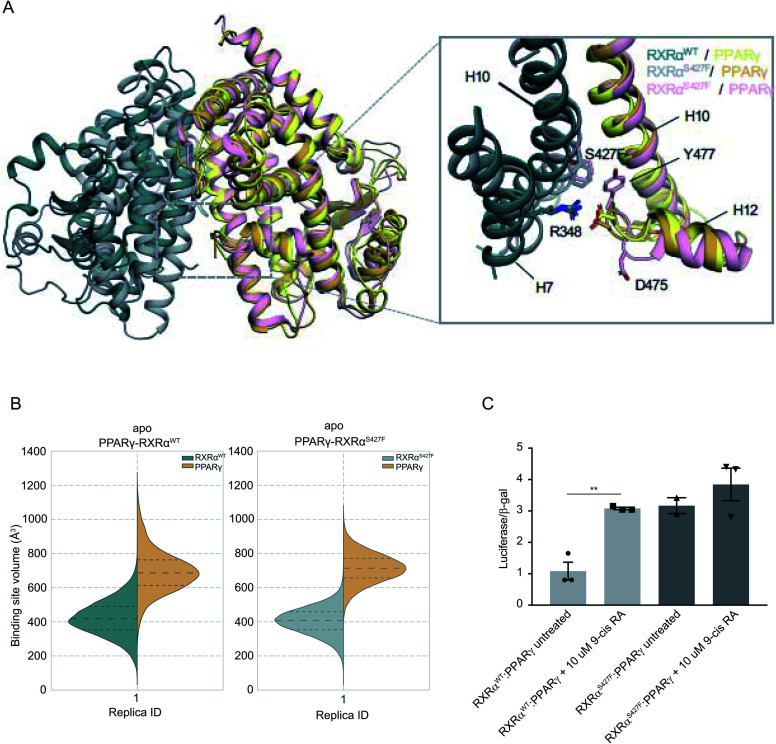
Computational and functional study of RXRα–PPARα heterodimers. (A) Effect of the RXRα S427F on the LBD of the RXRα–PPARγ heterodimer. The two main conformations that Tyr477 and Asp475 of PPARγ sample in the RXRα^S427F^–PPARγ simulations are shown in orange and pink respectively. (B) Violin plots showing the distribution of the volume of the binding-site of RXRα and PPARγ in over the course of the simulations (Table S1†). The dashed line in the middle indicates the median of the distribution, while the other two dashed lines represent the interquartile ranges. Each RXRα^WT^ monomer is colored with green, each RXRα^S427F^ monomer is colored in grey and each PPARγ monomer in yellow. (C) Boxplot indicating relative luciferase activity of the WT and mutant RXRα heterodimers with PPARγ in response to 9-cis RA. In the *Y* axis is shown the normalized activity relatively to cells transfected with the DR1 reporter alone. * indicates *p* value < 0.05; ** indicates *p* value < 0.01. Student's *t*-test was used.

H12 is a crucial helical component of the LBD of nuclear receptors because its ligand-induced repositioning creates the necessary surface for co-activator interaction and thereby generates the transcriptional activity. Therefore, the stabilization of its position in the active, co-activator-competent conformation through the direct interaction of Phe427 with Ty477 or through an indirect interaction of Phe427 with Asp475 *via* Arg348, is likely to tip the equilibrium of PPARγ towards active conformations rather than autoinhibitory or co-repressor-competent ones.

Sequence alignment of *RARA* with *PPARG* (Fig. S17†) shows that the C-terminal residue Tyr of *PPARG* corresponds to Glu in *RARA*, which would be unable to interact with Phe427 of RXRα. It is, therefore, not surprising that the effect of the S427F is not the same as the one seen when RXRα^S427F^ is in complex with RARα. Moreover, unlike RXRα^S427F^–RARα and RXRα^S427F^–RXRα^S427F^, the introduction of S427F mutation does not alter the size or shape of the ligand binding pockets of either RXRα or PPARγ within the same timescale of simulation (Fig. 3), highlighting yet another difference in the effect of the mutation with respect to different dimerization partners. It should be noted that the S427F does not seem to have a statistically significant effect on the curvature of RXRα^S427F^ H10 with respect to the WT (Fig. S6†). However, RXRα^S427F/WT^ H10 of RXRα–PPARγ exhibits the smallest curvature among all examined dimers. We reasoned that Gln444 and especially Gln451 of PPARγ H10, which interact with Arg426 and Glu434, respectively (Fig. S18†), bring H10 of the two monomers together and suppress any effect of the mutation on the curvature (Gln444 is replaced by Ser and Gln451 is replaced by Glu in both RXRα and RARα).

In agreement with published^7^ and our own simulations, our luciferase assays indeed support the hypothesis that the RXRα^S427F^–PPARγ heterodimer is constitutively active. As Fig. 3C indicates, the RXRα^S427F^–PPARγ heterodimer is transcriptionally at least 3× stronger than the RXRα^WT^–PPARγ one. Moreover, while 9-cis RA induces the activity of the RXRα^WT^–PPARγ by a factor of 3, its effect on the mutant counterpart is negligible. This ligand-independent activity of the RXRα^S427F^–PPARγ heterodimer might account for its presumptive oncogenic activity in human cancer.

## Discussion and conclusions

Retinoic acid signaling, and particularly RXRs, have gained renewed interest due to the identification of missense mutations in various cancer types, but more commonly in bladder cancer.^17^ Recent studies have focused on the hotspot mutation RXRα^S427^ and have elucidated its putative oncogenic role through heterodimerization with PPARα.^7,8^ RXRs, however, are obligatory partners for a number of nuclear receptors and can also form homodimers. By extending our studies to additional homodimeric and heterodimeric complexes (with RARα and PPARγ), we uncovered that the functional consequences of the S427F mutation are not universal.

To our knowledge, this is the first description of a naturally-occurring cancer-associated single amino acid substitution that affects the function of the mutant protein variably, depending on its dimerization partner. Our experimental data show that the RXRα S427F mutation leads to responsiveness to RAR but not RXR ligands in RXRα^S427F^–RARα heterodimers, implying a loss-of-function effect of RXRα^S427F^. On the other hand, the same mutation is gain-of-function independently of RXRα or PPARγ ligands in RXRα^S427F^–PPARγ heterodimers. Finally, RXRα^S427F^ behaves as a loss-of-function in response to RXR ligands in RXRα^S427^–RXRα^S427^ homodimers.

Our simulations rationalize the experimental findings for the three different dimers: (1) for the RXRα^S427F^–RARα heterodimer, the position of H11 of RXRα^S427F^ and consequently of H3 can be affected by the presence of the S427F mutation. The mutation-driven rotation of H3 towards the ligand-binding pocket abolishes, in turn, the ability of RXRα^S427^ to bind 9-cis RA or other ligands. (2) The gain of function phenotype of the mutant RXRα^S427F^–PPARγ heterodimer is manifested through the cooperative effect of two states that stabilize PPARγ H12 in the active conformation. In one state, the terminal Tyr477 of PPARγ interacts directly with Phe427 of RXRα^S427^, while in the other Asp475 of PPARγ H12 interacts with Arg348 of RXRα^S427^, which interacts with Phe427. In the RXRα–RXRα case, the stronger interactions of the RXRα^S427F^–RXRα^S427F^ and RXRα^WT^–RXRα^S427F^ complexes indicate that these dimers are in a more stable conformation compared to the RXRα^WT^–RXRα^WT^ one. Moreover, the volume change of the binding pocket in the RXRα^S427F^-containing complexes may affect favorable interactions with 9-cis RA rendering the complex less active, as indicated by functional assays. This was also confirmed from the docking calculations where one RXRα^S427F^ monomer was able to partially bind 9-cis RA in both RXRα^S427F^–RXRα^S427F^ and RXRα^WT^–RXRα^S427F^ dimers. Thus, the RXRα–RXRα homodimer demonstrates a completely different behaviour, concerning the binding of 9-cis RA, compared to the RXRα–PPARγ and RXRα–RARα heterodimers. These findings indicate that the S427F substitution, contrary to being thought to disrupt the dimer interface where it resides, rather affects allosterically the binding pocket.

A rather unexpected experimental finding was that RXRα^S427F^–RXRα^S427F^ and RXRα^WT^–RXRα^S427F^ dimers show comparable transcriptional activity implying that the mutation is detrimental in RXRα^WT^–RXRα^S427F^ dimers, which is not the case in the RXRα^S427F^–RARα heterodimers. It has been reported that the LBD of RXRα forms homodimers which are less stable than heterodimers with RARα, while mutations in the LBD region of RXRα close to S427 almost completely abolish RXRα homodimerization with no effect on heterodimers with RARα.^18^ Moreover, ligand binding is crucial for the stability of RXRα LBD homodimers.^16,39^ Therefore, the impact of the S427F mutation on the RXRα homodimers could be the result of both structural instability and reduced affinity to ligand binding due to pocket size changes.

RXRs are obligatory partners for a large number of nuclear receptors in various tissues.^17^ The S427F mutation is more frequently identified in bladder cancer, in which retinoic acid signaling plays important roles in tissue homeostasis.^19^ On the other hand, S427 RXRα mutations are also found in hepatocellular carcinoma and pancreatic adenocarcinoma^20^ in which the Liver X receptor (LXR) is the preferential partner of RXRs. It would be thus interesting to investigate how the same amino acid substitution affects the activity of RXRα heterodimers with LXR or any other partner, and the functional implications of this in tissue homeostasis and disease. Retinoids have been long used as chemotherapeutics and chemopreventives in several tissues, including the urinary bladder.^21^ This implies that activation of retinoic acid signaling suppresses tumorigenesis. On the other hand, expression data from bladder cancer tumors harboring RXRα mutations revealed increased expression of genes involved in adipogenesis and lipid metabolism, implying that S427 mutations cause constitutive activation. Moreover, genes upregulated in RXRα mutant, and particularly the RXRα^S427^ mutant tumors, are identified as PPARγ targets.^22^ Overall, these findings imply that RXRα^S427F^ mutant acts as an oncogene, a notion that contradicts the established role of retinoic signaling as a tumor suppressor. This apparent contradiction highlights the complex nature of nuclear receptor function which relies upon heterodimer formation. The RXR partner defines the target gene. Promoters containing DR1 sites can be bound by heterodimers with RARs, PPARs, HNF4, COUP-TF1 and RXR homodimers; DR2 is recognized by PPARs, RARs and RXRs; DR3 is bound by RXR heterodimers with vitamin D receptor (VDR); DR4 is targeted by RXR heterodimers with thyroid receptor (TR), LXR and RARs; and DR5 is recognized by heterodimers with RARs and Nurr77.^23^ It is safe to assume that the protein abundance of RXRs as well as of all candidate partners within cells will likely affect whether an RXRα mutation will confer a selective advantage in the process of neoplastic transformation. Moreover, the net functional outcome of RXRα^S427F^ could also be tuned by the effect of the mutation in the stability of each heterodimer. Our MD simulations showed that RXRα^S427F^-containing homodimers are more stable than the respective wt ones. This implies that the S427F mutation might entrap wt RXRα molecules in nonfunctional or partially functional dimers, thus depriving other nuclear receptors, such as PPARγ, of their obligatory dimerization partner.

This study opens up new avenues to fully elucidate the role of S427 RXRα mutations in human cancer and the mechanistic aspects of its involvement in tumorigenesis. This work is a significant step towards this goal because our results introduce for the first time the concept of pleiotropy of an individual single amino acid substitution depending on the structural and possibly the cellular context. Given the fact that all transcription factors act as homodimeric, heterodimeric or even supramolecular complexes with other transcriptional regulators, this work raises the intriguing possibility that these mechanistic insights apply to other transcription factors as well.

## Methods

### Expression of recombinant human RXRα protein in *Escherichia coli* (*E. coli*)

The coding region of the human RXRα^WT^ or RXRα^427F^ was subcloned into the pGEM-5X-1 vector. The BL21 + *E. coli* strain was used for protein expression. Single colonies were inoculated in 5 mL LB containing 100 μg mL^−1^ ampicillin overnight at 37 °C and used as inoculum for 500 mL of LB containing 100 μg mL^−1^ ampicillin. Bacterial cultures were incubated with shaking at 37 °C until they reached the mid-log phase of growth (A550 = 0.5–1.0). Protein expression was induced with 1 mM IPTG for 3 h. Bacteria were harvested with centrifugation at 5000*g* and cell pellets were further processed according to the protein purification protocol or kept at −20 °C until further use. GST-fused RXRα^WT^ and RXRα^S427F^ proteins were purified by affinity chromatography on Glutathione agarose using Protino® Glutathione Agarose 4B (Macherey–Nagel, REF. 745500.10) according to the manufacturer's protocol.

### Electrophoretic mobility shift assay (EMSA)

One hundred pmol of radiolabeled probe and 1 μL of 1 mg mL^−1^ poly(dI-dC) were mixed with recombinant RXRα proteins in binding buffer (10 mM Tris HCl pH 8.0, 150 mM KCl, 0.5 mM EDTA, 0.1% Triton X-100, 12.5% glycerol, 0.2 mM DTT). The final volume of the reaction was 20 μL. The binding reactions were incubated for 10 minutes at RT and 5 minutes at 37 °C. The DNA–protein complexes were run on 5% polyacrylamide gels in 0.5× TBE at 200 V. Polyacrylamide gels were pre-run at 200 V for 2 hours. Both pre-running and DNA–protein complexes electrophoresis were performed at 4 °C. After the electrophoresis was completed, the gel was transferred to Whatman paper, covered with a plastic membrane and dried at 80 °C in a vacuum dryer for 1–2 hours. The dried gel was exposed to an autoradiograph film (Fujifilm) at −80 °C for 1–2 hours.

### EMSA probe generation and labeling

To generate DR1-containing and control (half-site) probes for EMSA, we annealed the following oligonucleotides:

Name sequence

DR1_F: TCGAGGGTAGGGGTCAGAGGTCACTCGTCGA

DR1_R: TCGACGAGTGACCTCTGACCCCTACCCTCGA

Half-site_F: AGCTTGGCGCCAGGGGTCAGGTCAGAATT

Half-site_R: AATTCTGACCTGACCCCTGGCGCCAAGCT

Prior to annealing, the forward (F) oligonucleotide was radiolabeled with T4 polynucleotide kinase (New England Biolabs) according to the vendor's instructions.

### Luciferase reporter assays

For luciferase assays, 3 × 10^5^ HEK293T were transfected with 500 ng of a reporter plasmid containing the luciferase gene under the control of DR1 or DR5 response elements. In experiments using the DR1-luc reporter, cells were co-transfected with a modified pLKO.1/IRESegfp vector expressing either RXRα^WT^ or RXRα^S427F^ under the control of a CMV promoter. In experiments using the DR5-luc reporter, cells were co-transfected with equal amounts of a pLKO.1/IRESegfp expressing either RXRα^WT^ or RXRα^S427F^ and RARα. In all experiments a plasmid expressing β-galactosidase was co-transfected in tracer amounts. Twelve hours after transfection fresh medium supplemented with RXRα or RARα agonists was added. Cells were harvested 48 hours post transfection for luciferase assays and β-galactosidase colorimetric assays.

### DNA binding properties of RXRα^S427F^

To assess the effect of the S427F mutation on DNA binding, we expressed RXRα^427F^ and RXRα^WT^ in *E. coli* bacteria and purified the recombinant proteins. We used those in EMSA experiments with radiolabeled DR1-containing oligonucleotides. As Fig. S2† indicates, both mutant and WT proteins bind the DR1 site with the same efficiency. In fact, we observe two different band shifts (shifts 1 and 2) possibly corresponding to dimeric and tetrameric complexes of RXRα. Bands disappear upon competition with unlabeled DR-containing oligonucleotides implying that the observed binding is absolutely specific.

### MD simulations

We performed a series of molecular dynamics (MD) simulations under different mutation and ligated conditions. A list of all the performed simulations, along with the simulation time of each is given in Table S1.† The atomistic model of the LBD–LBD of the RXRα–RXRα homodimer was based on the crystal structure of the human RXRα LBD bound to the rexinoid agonist BMS649 (PDB ID 1MVC),^24^ of the RXRα–RARα heterodimer on the crystal structure of the human RARα LBD bound to its natural agonist all-trans retinoic acid (atRA), and the mouse RXRα LBD bound to the rexinoid antagonist LG10074 (PDB ID 3A9E).^25^ Modeling of LBD–LBD of the RXRα–PPARγ heterodimer was based on the crystal structure of the human RXRα LBD bound to its natural agonist 9-cis RA, and the human PPARγ LBD bound to the agonist rosiglitazone (PDB ID 1FM6).^26^ Full details of protein modeling, protonation states, *etc.*, can be found in the ESI.† Atomic charges for the at-RA, 9-cis RA, and LG100754 ligands were calculated by restrained electrostatic potential (RESP) fitting (RESP-A1A mode) at the Hartree–Fock level with a 6-31G* basis set, as applied in the R.E.D. IV server version 3.0.^27–29^ The general amber force field (GAFF)^30^ was used for the bonded and non-bonded interactions of the ligands. The GROMACS v5.0.7 MD engine^31^ was used for the simulations of the RXRα–RARα complexes, while GROMACS v.2018.6 was used for the simulations of the RXRα–PPARγ complexes and GROMACS v.2020.4 was used for the simulations of the RXRα–RXRα complexes. We used the amber 99SB*-ILDN force field^32^ to describe the protein dynamics of the RXRα–RXRα and RXRα–RARα complexes, and the Amber03 force field^33^ for the RXRα–PPARγ in accordance with the force field that was used by Halstead *et al.*^7^ Prior to MD simulations, all structures were subjected to 10 000 steps of energy minimization using the steepest descent algorithm, followed by position restraint equilibration first in the NVT and then in the NPT ensemble for 150 ps, respectively. Once equilibrated at constant pressure, unbiased MD simulations were carried out in the canonical ensemble (NPT) with the atomic coordinates of the system saved every 10 ps. Production runs range from 370 ns to 2 μs and were performed in three replicas each for the apo systems and one replica for the holo systems. Long-range electrostatic interactions were treated using the particle-mesh Ewald scheme^33^ with a grid spacing of 1.6 Å, while a cut-off 10 Å was applied for the van der Waals interactions. All bonds were constrained using the LINCS algorithm allowing for a time-step of 2 fs. The non-bonded potential energy functions were switched, with forces decaying between 0.8 and 1.0 nm. The Parrinello–Rahman barostat^34^ maintained a target pressure of 1 bar isotropically with a time constant of *τ*_P_ = 2 ps and compressibility of 4.5 × 10^−5^ bar^−1^, while the Nosé–Hoover thermostat^35^ was applied throughout all the simulations to keep the temperature at 310 K using a coupling constant of *τ*_T_ = 0.5 ps. To obtain the representative structure of the equilibrated RXRα–RARα and RXRα–PPARγ dimeric complexes, we clustered the conformations of each complex during the last 100 ns of each simulation, while we clustered the conformations of each RXRα–RXRα complex during the last 500 ns of each simulation. For this purpose, the GROMOS algorithm^36^ of the gmx_cluster routine (GROMACS) was used. The natural RXRα agonist 9-cis RA was docked into both receptors of the RXRα–RARα and RXRα–RXRα representative structures using the default protocol of the Glide – Ligand Docking tool of the Schrödinger suite (Schrödinger, LLC, New York, NY, 2020). The natural RARα agonist atRA was docked into RARa receptor of the RXRα–RARα representative structures using the same docking protocol. The Epock VMD plug-in ref. 37 was used to calculate the accessible volume of the RXRα, RARα and PPARγ binding sites. Kink Finder^38^ was used to measure angles in helices. The NetworkView VMD plug-in was used to perform the Dynamical Network Analysis method in order to construct network models obtained from our MD simulations.^37^

## Author contributions

I. G., M. P., P. K. and G. S. performed computational experiments. V. B. and P. V. performed molecular biology experiments. I. G., V. B., M. P., Z. C. and A. K. wrote and revised the manuscript. Z. C. and A. K. conceived the study and supervised the research.

## Conflicts of interest

There are no conflicts of interest to declare.

## Supplementary Material

SC-012-D1SC04465F-s001

## Data Availability

The datasets/input/output files generated during this study are available at http://dx.doi.org/10.5281/zenodo.5534571.
