# Supplementary material for: The effect of S427F mutation on RXRα activity depends on its dimeric partner
Source: Chem Sci. 2021 Oct 8;12(44):14700–10. doi: 10.1039/d1sc04465f (PMC8597827; doi:10.1039/d1sc04465f)
Supplement: SC-012-D1SC04465F-s001 [file SC-012-D1SC04465F-s001.pdf]

Electronic Supplementary Material (ESI) for Chemical Science.
This journal is © The Royal Society of Chemistry 2021

SUPPLEMENTAL INFORMATION

The effect of S427F mutation on RXRα activity depends on its dimeric partner

Ioannis Galdadas[1‡], Evangelos Bonis[1‡], Paraskevi Vgenopoulou[1‡], Michail Papadourakis[1], Panos Kakoulidis[1,2], Georgia Stergiou[1,2], Zoe Cournia[1,2,], Apostolos Klinakis[1,]**

[1] Biomedical Research Foundation Academy of Athens, Athens, Greece.

[2] Data Science and Information Technologies, Department of Informatics and Telecommunication, National and Kapodistrian University of Athens, Athens, Greece.

1. MD simulations - Protein structures preparation

To better understand the effect of the S427F mutation on the structure and dynamics of the ligand binding domain (LBD) of RXRα when in complex with the LBD of RXRα, RARα, or PPARγ, we performed a series of independent molecular dynamics (MD) simulations under different mutation and ligated conditions. A list of all the performed simulations, along with the simulation time of each is given in Table S1.

RXRα-RXRα homodimer

The atomistic model of the LBD-LBD of the RXRα-RXRα homodimer was based on the crystal structure of the human RXRα LBD bound to the rexinoid agonist BMS649 (PDB ID 1MVC)[1]. To model the unresolved region Lys245-Asn262 of each RXRα monomer in the crystal structure, we used the Prime 4.2 tool of the Schrödinger suite (Schrödinger, LLC, New York, NY, 2015)[2,3]. Unresolved residues that correspond to highly flexible regions located at the C- and N-termini of the monomers were not modeled since their conformation could not be predicted with accuracy.

The protonation state of each residue was determined using the PROPKA3.0 algorithm[4] at pH 7, which assigned the usual charge states to all residues. The δ-nitrogen was protonated for residues His331, 338, and 406. We left all chain termini uncapped.

The co-crystalized agonist and any co-crystalized water molecule beyond 5 Å away from the protein surface were removed, and the natural RXRα agonist 9-cis retinoic acid (9-cis RA) was docked into both receptors using the default protocol of the Glide – Ligand Docking tool of the Schrödinger suite (Schrödinger, LLC, New York, NY, 2015), (holo simulations section, Table S1). The ligands, as well as the co-crystalized water molecules, were removed in the RXRα-RXRα complex (apo simulations section, Table S1). The co-crystalized coactivator peptide was also removed in both the apo and holo systems.

RXRα-RARα heterodimer

The atomistic model of the LBD-LBD of the RXRα-RARα heterodimer was based on the crystal structure of the human RARα LBD bound to its natural agonist all-trans retinoic acid (atRA), and the mouse RXRα LBD bound to the rexinoid antagonist LG10074 (PDB ID 3A9E)[5]. Given the high identity percentage of the human and mouse RXRα sequences (>99% as calculated through BLAST[6]), we are confident that our findings can be extended to the human RXRα-RARα heterodimer as well. The numbering of the residues of RXRα in the RXRα-RARα crystal structure was shifted such that the mutated serine is located at position 427. The co-activator peptide TIF-2 that was bound to the activation function-2 (AF-2) surface of the active state of RARα in the crystal structure was kept in all of the MD simulations of the RXRα-RARα complex. To model the unresolved region Thr251-Ser264 of RXRα in the RXRα-RARα crystal structure, we used the Prime 4.2 tool of the Schrödinger suite (Schrödinger, LLC, New York, NY, 2015)[2,3]. Unresolved residues that correspond to highly flexible regions located at the C- and N-termini of the monomers were not modeled since their conformation could not be predicted with accuracy. The co-crystalized water molecule in the vicinity of LG100754 that has been reported by Sato et al.[5] to be involved in a hydrogen bond network between the carboxyl group of LG100754 and the amino group of Leu309 was also kept to its crystallographic position in the initial conformations of the simulations of the RARα/atRA-RXRα/LG100754 complex (holo simulations section, Table S1). The ligands, as well as the co-crystalized water molecules were removed in the RARα-RXRα complex (apo simulations section, Table S1).

The protonation state of each residue was determined using the PROPKA3.0 algorithm[4] at pH 7, which assigned the usual charge states to all residues, except for RXRα:Glu394, Glu247, which were predicted to be protonated. The δ-nitrogen was protonated for residues RXRα:His331 and

RARα:His190, His293, His367, while a p-protonation (both δ and ε protonations), was assigned for RXRα:His288, His338. We left all chain termini uncapped.

RXRα-RPARγ heterodimer

The atomistic model of the LBD-LBD of the RXRα-PPARγ heterodimer was based on the crystal structure of the human RXRα LBD bound to its natural agonist 9-cis RA, and the human PPARγ LBD bound to the agonist rosiglitazone (PDB ID 1FM6).[7] The co-crystalized ligands, water molecules, as well as the co-activator peptide NCOA1 in the activation-function 2 (AF-2) domain were not included in our simulations (apo simulations section, Table S1) so that our results can be compared with the results reported by Halstead et al.[8], where the co-crystalized ligands and the co-activator peptides were removed to reduce the computational complexity and allow a potential transition to an inactive state during simulations.

The protonation state of each residue was determined using the PROPKA3.0 algorithm[4] at pH 7, which assigned the usual charge states to all residues. The δ-nitrogen was protonated for residues RXRα:His288, 315, 331, and 406. We left all chain termini uncapped.

We introduced the single point mutation, S427F, in all of the mutant RXRα-RARα, RXRα-RXRα, and RXRα-PPARγ complexes using Maestro 15.4 (Schrödinger LCC) software. The optimum orientation of the side-chain of Phe after the mutation was predicted in each complex using the "Side-chain Prediction" tool of Prime 4.2.

Prior to simulations, we minimized the potential energy of the prepared complexes using the OPLS3 force-field[9], and we solvated the complexes with explicit TIP3P[10] water molecules in a cubic box with periodic boundary conditions. The size of the box was such that ensured a minimum distance of 1.5 nm between the proteins and the edge of the simulation box. Sodium counterions were added to neutralize the net charges of each system, respectively.

Ligand Parameterization

To investigate the effect of the presence of ligands to the behavior of the RXRα-RXRα homodimer and RXRα-RARα heterodimer, we performed simulations of the wild-type and mutant complexes in the presence of the co-crystallized RXRα antagonist LG100754, the natural RXRα agonist 9-cis RA, and the co-crystallized RARα agonist atRA (holo simulations section, Table S1).

The atomic charges for the atRA, 9-cis RA, and LG100754 were calculated by restrained electrostatic potential (RESP) fitting (RESP-A1A mode) at the Hartee-Fock level with a 6-31G* basis set, as applied in the R.E.D. IV server version 3.0.[11–13] The general Amber force field (GAFF)[14] was used for the bonded and non-bonded interactions of the ligands, and these parameters were combined with the quantum-mechanics-derived atomic charges using Antechamber as implemented in the CcpNmr ACPYPE too.l[15] The validity of the parameters for the ligands was checked through a 20 ns MD simulation in vacuo in the NVE ensemble, where we ensured that the molecules maintained the right geometry.

2. MD simulations - Simulation set-up

The GROMACS v5.0.7 MD engine[16] was used for the simulations of the RXRα-RARα complexes, while GROMACS v.2018.6 was used for the simulations of the RXRα-PPARγ complexes and GROMACS v.2020.4 was used for the simulations of the RXRα-RXRα. We used the Amber 99SB*-ILDN force field[17] to describe the protein dynamics of the RXRα-RXRα and RXRα-RARα complexes, and the Amber03 force field[18] for the RXRα-PPARγ in accordance with the force field that was used by Halstead et al.[8]

Prior to MD simulations, all structures were subjected to 10,000 steps of energy minimization using the steepest descent algorithm to remove possible atomic clashes and inconsistencies in the starting structures in terms of geometry and solvent orientation, followed by position restraint equilibration first in the NVT and then in the NPT ensemble for 150 ps, respectively. Once equilibrated at constant pressure, unbiased MD simulations were carried out in the canonical ensemble (NPT) with the atomic coordinates of the system saved every 10 ps.

Long-range electrostatic interactions were treated using the particle-mesh Ewald scheme[19] with a grid spacing of 1.6 Å, while a cut-off 10 Å was applied for the Van der Waals interactions. All bonds were constrained using the LINCS algorithm allowing for a time-step of 2 fs. The non-bonded potential energy functions were switched, with forces decaying between 0.8 and 1.0 nm. The Parrinello−Rahman barostat[20] maintained a target pressure of 1 bar isotropically with a time constant of τ_P =2 ps and compressibility of 4.5×10^{-5} bar^{-1}, while the Nosé-Hoover thermostat[21] was applied throughout all the simulations to keep the temperature at 310 K using a coupling constant of τ_T = 0.5 ps.

In replica systems, different initial velocities were generated from a Maxwell distribution with a random seed. According to the timeseries of the volume of the binding site of the mutant RXRα in the RXRα-RARα complex, the mutant-induced closure of the RXRα binding site takes place within the first 100 ns. Therefore, we run replica systems for 370 ns each, as such timescale is sufficient to monitor the motions that are related to the closure of the binding site.

Table S1. List of simulated RXRα-RXRα, RXRα-RARα, and RXRα-PPARγ dimers. coA: coactivator peptide (SRC-1) bound in the AF2 groove.

Liganted status	Monomer A	Monomer B	Replica ID: Simulation Time (ns)
apo	RXRα^{WT}	RXRα^{WT}	rep1: 2000<br>rep2: 2000<br>rep3: 2000
	RXRα^{S427F}	RXRα^{WT}	rep1: 2000<br>rep2: 2000<br>rep3: 2000
	RXRα^{S427F}	RXRα^{S427F}	rep1: 2000<br>rep2: 2000<br>rep3: 2000
	RXRα^{WT}	RARα^{WT} + SRC-1 coA	rep1: 925<br>rep2: 370<br>rep3: 370
	RXRα^{S427F}	RARα^{WT} + SRC-1 coA	rep1: 925<br>rep2: 370<br>rep3: 370
	RXRα^{WT}	PPARγ^{WT}	rep1: 370
	RXRα^{S427F}	PPARγ^{WT}	rep1: 370
holo	RXRα^{WT} + 9-cis RA (agonist)	RXRα^{WT} + 9-cis RA (agonist)	rep1: 370

RXRα^{WT} + 9-cis RA (agonist)	RXRα^{S427F} + 9-cis RA (agonist)	rep1: 370
RXRα^{S427F} + 9-cis RA (agonist)	RXRα^{S427F} + 9-cis RA (agonist)	rep1: 370
RXRα^{WT} + LG100754 (antagonist)	RARα^{WT} + SRC-1 coA + atRA (agonist)	rep1: 925
RXRα^{S427F} + LG100754 (antagonist)	RARα^{WT} + SRC-1 coA + atRA (agonist)	rep1: 925

3. MD simulations - Representative structures

To obtain the representative structure of the equilibrated RXRα-RARα and RXRα-PPARγ dimeric complexes, we clustered the conformations of each complex during the last 100 ns of each simulation, while we clustered the conformations of each RXRα-RXRα complex during the last 500 ns of each simulation. The *gromos* algorithm[22] of the gmx_cluster routine (GROMACS) was used with the root mean squared deviation (RMSD) clustering criterion on the C_α atoms as the distance between the structures and a cutoff value of 1.75 Å. The cutoff value was chosen so that to obtain balanced cluster sizes. In the most populated cluster, the central structure, i.e., the structure with the smallest distance to all of the other members of the cluster, was picked as the representative of the equilibrated system.

4. MD simulations - RXRα binding site volume

We used the Epock VMD-plug in[23] to calculate the accessible volume of the RXRα, RARα and PPARγ binding sites. We described the binding site pocket of RXRα and RARα as the space defined by the Cα atoms of the residues that lie within 5 Å away from the LG100754 and atRA ligands respectively in the RXRα/LG100754 - RARα/atRA crystal structure.[5] The same residues were used for consistency to define the binding site pocket of RXRα in the homodimeric form of RXRα, and the pocket in complex with PPARγ. Regarding the binding site of PPARγ, we used the Cα atoms of the PPARγ residues that lie within 5 Å away from the bound ligand rosiglitazone in

the RXRα/9-cis RA - PPARγ/rosiglitazone crystal structure.[7] For the calculation, the protein grid mesh was created using a grid distance of 0.5 Å, as a lower distance has been reported to increase the execution time exponentially without improving the accuracy of the calculation. The probe radius for free space detection was set to 1.2 Å.

5. MD simulations - RXRα H10 kink

Kink Finder[23] was used to measure angles in helices. Kink Finder fits a cylinder to every 6-residue segment of a helix by minimizing r,

$$r = \sqrt{\frac{1}{m}\sum_{i=1}^{m}(d_i - \bar{d})^2}$$

Where m is the number of backbone atoms in the segment, d_i is the shortest distance from backbone atom i to the fitted helix axis, and $\bar{d}$ is the mean of all distances. The calculation of a kink angle is done by first defining two-cylinder fits, one for the residues N-terminal of the kink position, and one for the set of six residues C-terminal to the kink. The angle of the kink is then is measured between the axes of the two cylinders. In our case, the kink position was predicted by Kink Finder to lie at position 422 (Leu422) of H10 of RXRα, right before Pro423, and the six residues before and after that position were used to fit the two cylinders for the calculation of the king angle over the course of the simulations.

6. MD simulations – Dynamical Network Analysis

The NetworkView VMD plug-in was used to perform the Dynamical Network Analysis method to construct network models obtained from our MD simulations. Through this method, the intramolecular interactions in a protein can be collectively represented in the form of a network, which is defined as a set of nodes (residues) with connected edges (links) that depend on the node interaction strength. This network can then elucidate potential allosteric regulation within a given protein. Apart from identifying allosteric pathways, the method also produces shortest paths between residues as the most dominant mode of their communication.

In our study, to create a network model for our protein, the nodes and edges need to be created using a similar protocol as in Sethi et al[24]. We used as nodes the Cα atoms of each RXRα residue. We chose to connect through edges only the nodes that are within a cutoff distance of 4.5 Å of another residue for at least 75% of the trajectory Namely, if a residue i is within 4.5 Å of another residue j for 75% of the MD simulation, a weight is assigned to their edge that defines the probability of information transfer across that edge; otherwise, the weight was set to zero.
Finally, it is also important to identify residues that play a key role in transmitting the signal from one region of a protein to another. For this purpose, we define the path through which the signal is transmitted between the source of the allosteric signal (Trp305 of one RXRα monomer) and the end-point of the allosteric signal (Trp305 of the other RXRα monomer). According to del Sol et al. (2006), nodes that are key in preserving short paths in network communication, are crucial for the transmission of the signal. The shortest (optimal) path between two nodes in a network is determined using the Floyd-Warshall algorithm.

In our calculations, these analyses have been applied for the last 500 ns of each simulation run.

7. DNA binding properties of RXRα^{S427F}

To assess the effect of the S427F mutation on DNA binding, we expressed RXRα^{427F} and RXRα^{WT} in E. coli bacteria and purified the recombinant proteins. We used those in EMSA experiments with radiolabeled DR1-containing oligonucleotides. As Figure S2 indicates, both mutant and WT proteins bind the DR1 site with the same efficiency. In fact, we observe two different band shifts (shifts 1 and 2) possibly corresponding to dimeric and tetrameric complexes of RXRα. Bands disappear upon competition with unlabeled DR-containing oligonucleotides implying that the observed binding is absolutely specific.

Table S2. List of predicted binding free energies of 9-cis RA to each RXRα and RARα monomer and atRA to each RARα monomer obtained from docking calculations.

**Replica**	**Monomer A**	**Docking score 9-cis (kcal/mol)**	**Monomer B**	**Docking score 9-cis (kcal/mol)**	**Docking score atRA (kcal/mol)**
1	RXRα^{WT}	-4.65	RARα	-6.73	-7.35
2	RXRα^{WT}	-6.62	RARα	-6.68	-8.68
3	RXRα^{WT}	-6.65	RARα	-6.12	-7.91
1	RXRα^{S427F}	>0	RARα	-5.55	-5.28
2	RXRα^{S427F}	>0	RARα	-7.64	-6.65
3	RXRα^{S427F}	-0.60	RARα	-3.93	-4.90

Table S3. List of average volume of the binding pocket for each RXRα monomer obtained from the MD simulations of the three replicas.

**Monomer A**	**Volume (Å^3)**	**Monomer B**	**Volume (Å^3)**
RXRα^{WT}	199.77±118.45	RXRα^{WT}	203.91±118.91
RXRα^{S427F}	152.79±91.14	RXRα^{S427F}	287.57±129.45
RXRα^{S427F}	249.52±104.62	RXRα^{WT}	139.70±80.22

Table S4. List of the average volume of the binding pocket for each RXRα monomer obtained from the MD simulations.

Replica	Monomer A	Volume (Å^3)	Monomer B	Volume (Å^3)
1	RXRα^{WT}	179.59±55.89	RXRα^{WT}	193.33±51.94
2	RXRα^{WT}	288.40±91.14	RXRα^{WT}	292.66±102.03
3	RXRα^{WT}	131.32±51.00	RXRα^{WT}	125.73±31.15
1	RXRα^{S427F}	114.07±50.16	RXRα^{S427F}	267.37±83.26
2	RXRα^{S427F}	182.97±63.14	RXRα^{S427F}	259.90±76.49
3	RXRα^{S427F}	161.34±52.10	RXRα^{S427F}	335.43±63.05
1	RXRα^{S427F}	248.73±65.05	RXRα^{WT}	96.05±35.79
2	RXRα^{S427F}	259.61±49.93	RXRα^{WT}	140.43±50.29
3	RXRα^{S427F}	240.23±64.97	RXRα^{WT}	182.61±51.24

Table S5. List of predicted binding free energies of 9-cis RA to each RXRα monomer obtained from docking calculations.

Replica	Monomer A	Docking score (kcal/mol)	Monomer B	Docking score (kcal/mol)
1	RXRα^{WT}	-7.44	RXRα^{WT}	-7.65
2	RXRα^{WT}	-6.54	RXRα^{WT}	-7.92
3	RXRα^{WT}	-4.99	RXRα^{WT}	-8.60

1	RXRα^{S427F}	-2.07	RXRα^{S427F}	>0
2	RXRα^{S427F}	-1.64	RXRα^{S427F}	>0
3	RXRα^{S427F}	>0	RXRα^{S427F}	>0
1	RXRα^{S427F}	>0	RXRα^{WT}	-4.17
2	RXRα^{S427F}	>0	RXRα^{WT}	-3.56
3	RXRα^{S427F}	>0	RXRα^{WT}	-3.53

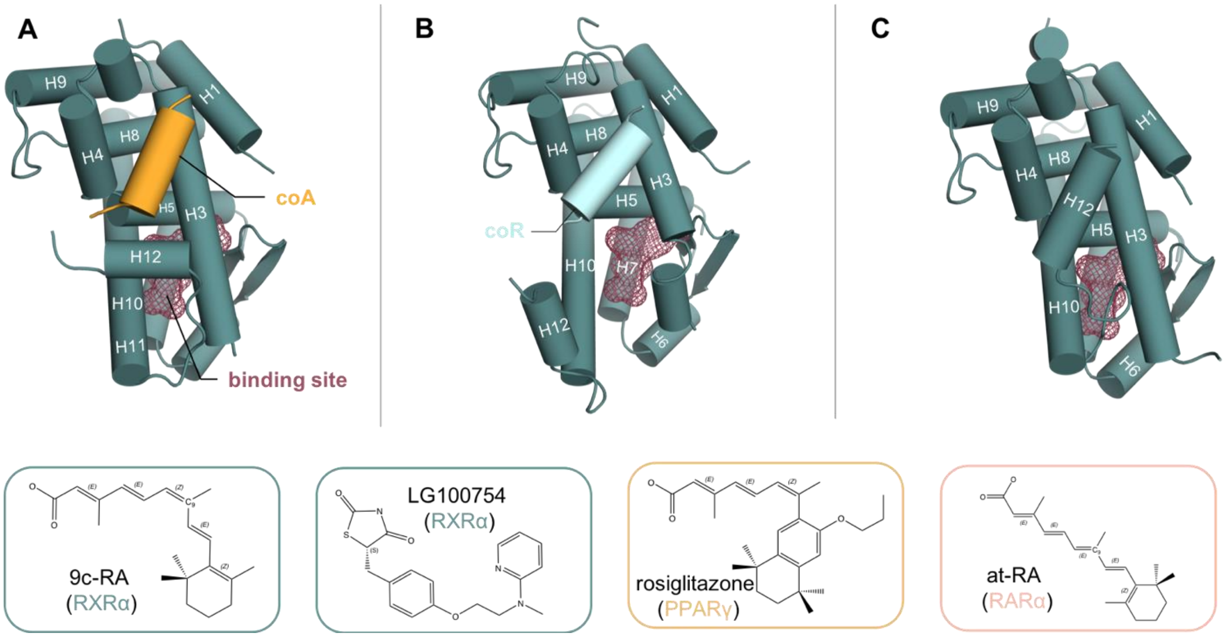

Figure S1. Cylinder representation of the overall structure of the LBD of RXRα in different states. The twelve helices (H1:H12) that define the receptor are numbered from N- to C-terminus. (A) active conformation (PDB ID 1MVC)[1] with a coactivator peptide (coA, orange) bound to the coactivator cleft generated by H3, H4 and H12, (B) inactive conformation (PDB ID 3R29)[24] with a corepressor peptide (coR, cyan) bound to the cleft, (C) inactive conformation (PDB ID 1DKF)[25] with H12 bound to the cleft. The chemical structures of the RXRα, RARα, and PPARγ ligands used in the simulations (Table S1) are depicted in the boxes underneath.

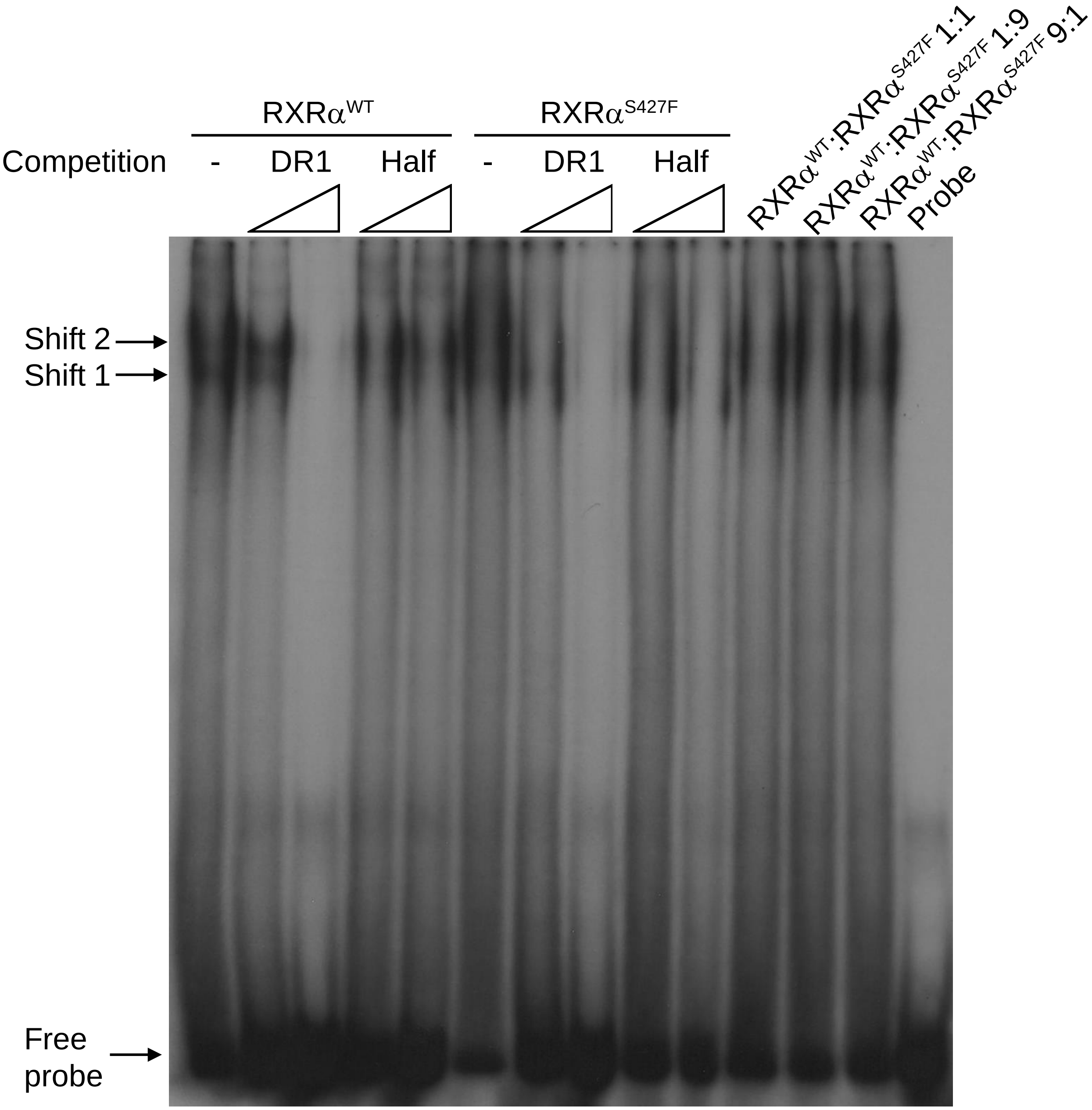

Figure S2. Radiograph of a representative EMSA experiment with mutant and wild-type RXRα with an oligonucleotide containing a DR1 site. Competition was performed with 5X and 25X excess of the indicated sequences. Note that unlabeled DR1 oligonucleotides compete in a dose-dependent manner the radiolabeled DR1 eliminating the observed shift, while half sites, which cannot be bound by RXRα, cannot compete at all. Identical results were obtained when WT or mutant RXRα were used alone or in combination with one another in two different ratios.

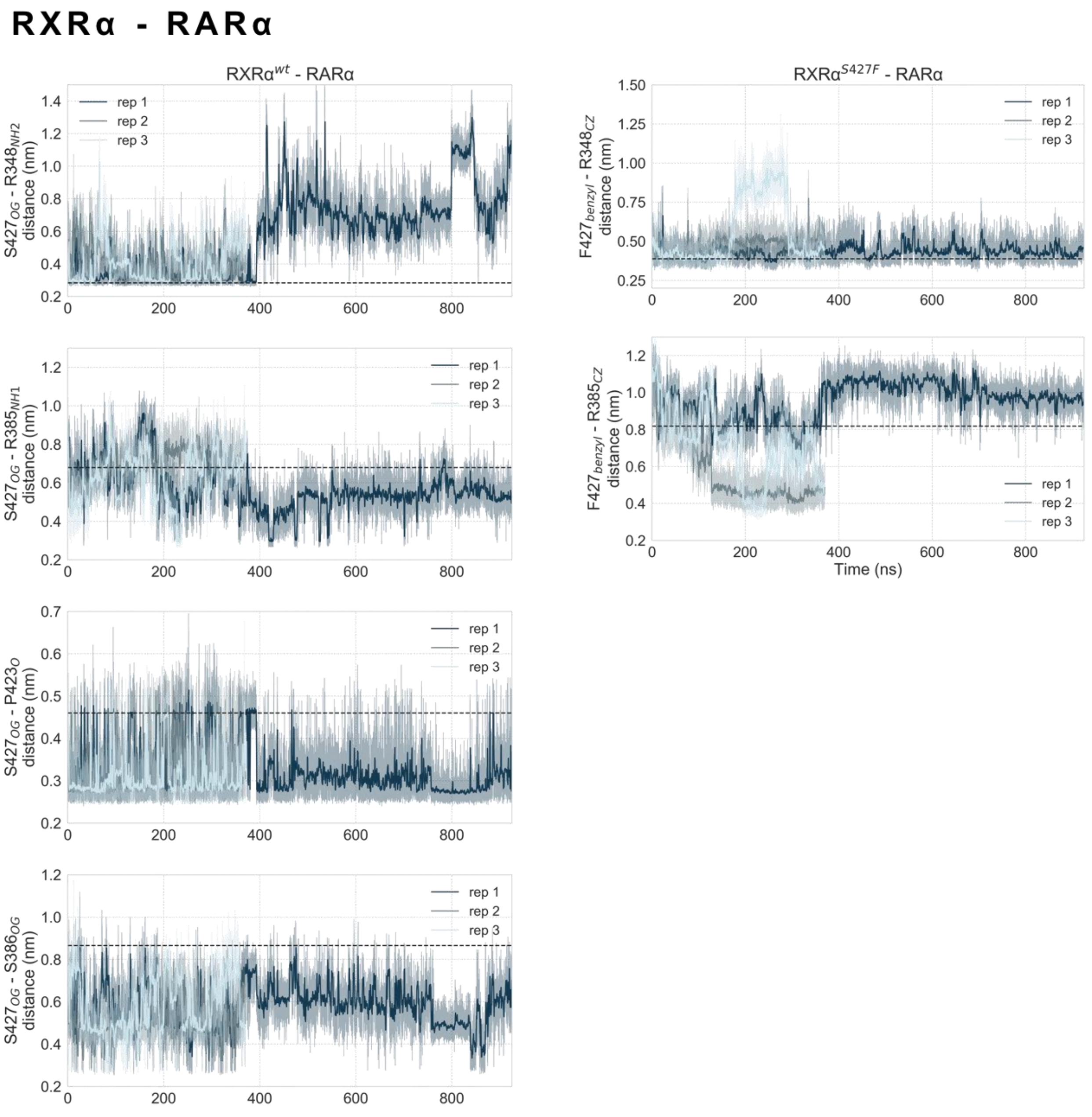

Figure S3. Time series of the Ser427 and Phe427 interactions in apo RXRα^{WT}-RARα and apo RXRα^{S427F}-RXRα. Time series of the interactions that Ser427$_{(OG)}$ and Phe427$_{(centre\ of\ mass\ of\ benzyl\ ring)}$ engage in over the course of the simulations of the apo RXRα^{wt}-RARα and apo RXRα^{S427F}-RXRα. Residues Arg348$_{(CZ)}$ and Pro423$_{(O)}$ belong to RXRα, while residues Ser386$_{(OG)}$ and Arg385$_{(NH1\ or\ CZ)}$ belong to RARα. Thick traces represent moving averages, whereas thin traces represent original, unsmoothed values. Dashed horizontal lines indicate distance values in the corresponding crystal structures (PDB ID 3A9E).

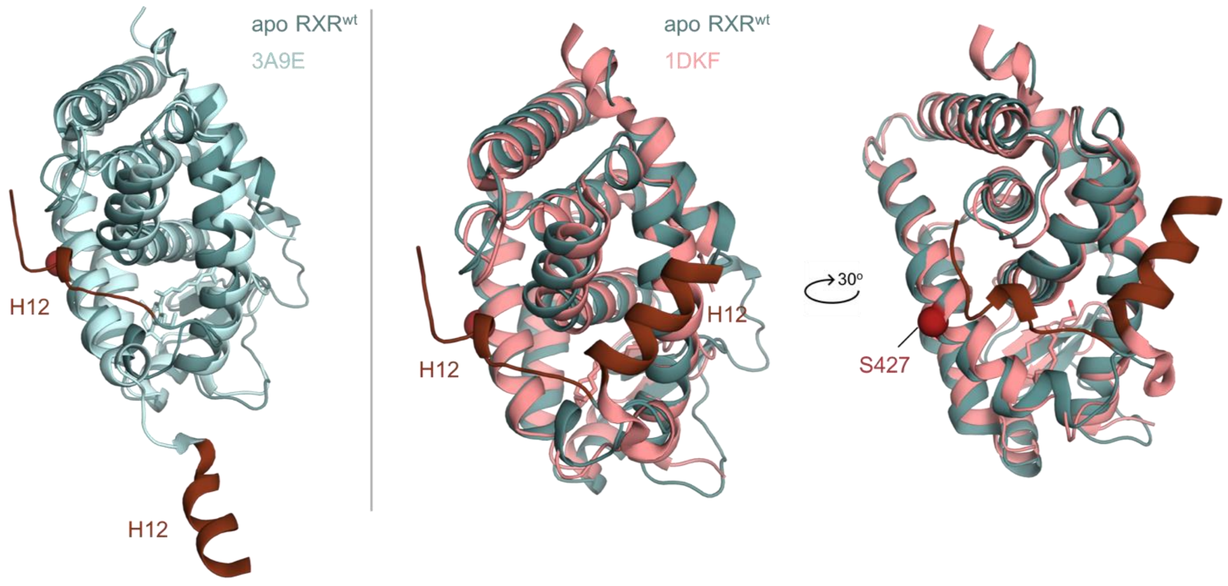

Figure S4. Comparison of the most frequently sampled conformation of the apo RXRα^{WT} from the RXRα^{WT}-RARα simulations, with the crystal structure of the RXRα^{WT} bound to the antagonist, LG100754. Comparison of the most frequently sampled conformation of the apo RXRα^{WT} (dark green) from the RXRα^{WT}-RARα simulations, with the crystal structure of the RXRα^{WT} bound to the antagonist, LG100754 (cyan, PDB ID 3A9E), and with the crystal structure of the RXRα^{WT} bound to the agonist, oleic acid (pink, PDB ID 1DKF). The simulations started from the antagonist conformation where H12 (brown) points to the solvent and soon converge towards an antagonist-like conformation where H12 binds to the co-factor binding site. The position of the S427F mutation is depicted through a red sphere.

A

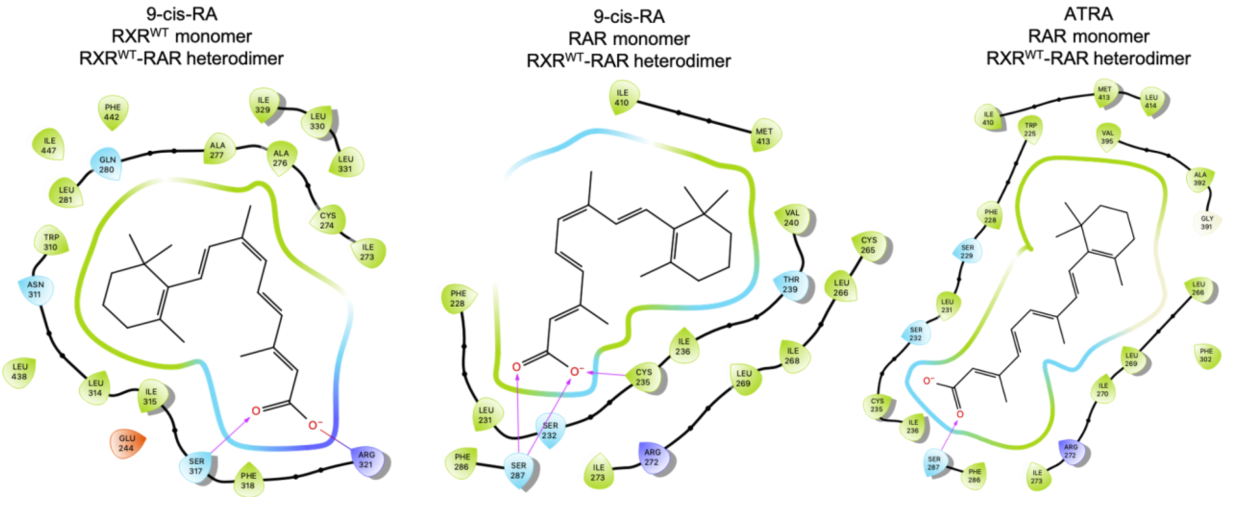

B

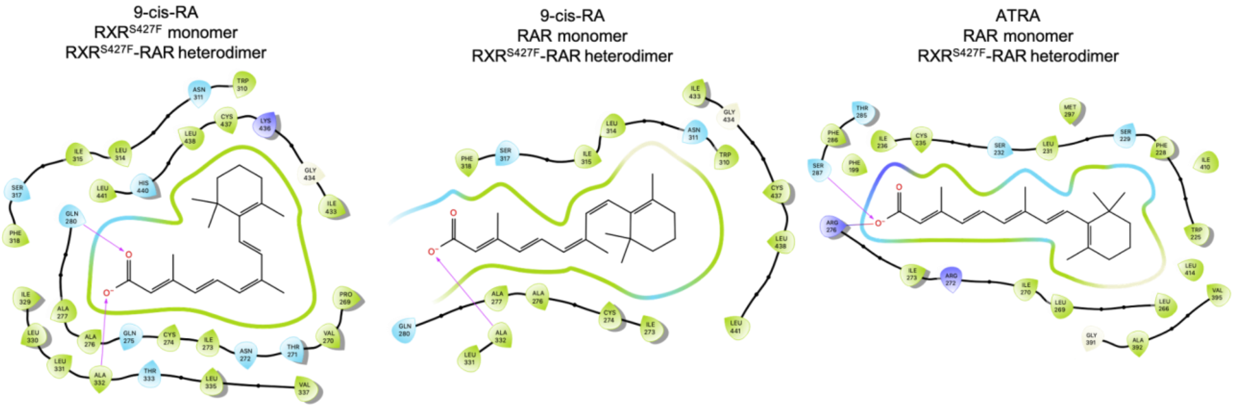

Figure S5. Ligand Interaction diagram of 9-cis and atRA bound to each RXRα and RARα monomer of the RXRα^{WT}-RXRα^{WT}, RXRα^{S427F}-RXRα^{S427F} and RXRα^{S427F}-RXRα^{WT} homodimers.

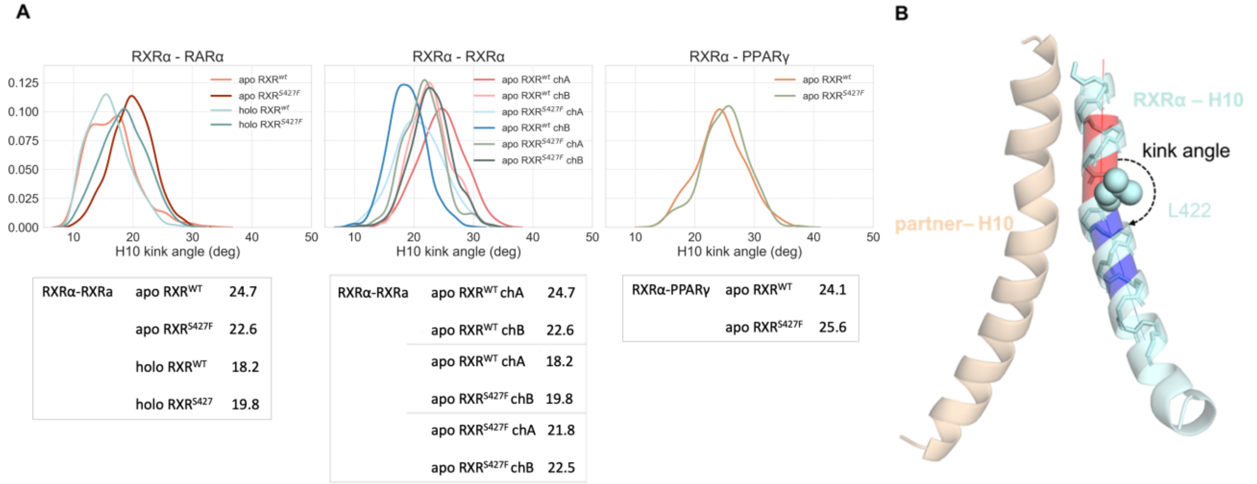

Figure S6. Distribution of the RXRα H10 kink angle in the RXRα-RARα, RXRα-RXRα, and RXRα-PPARγ dimers over the course of the simulations. (A) Distribution of the RXRα H10 kink angle in the RXRα-RARα, RXRα-RXRα, and RXR-PPAR**γ** dimers over the course of the simulations. In the case of RXRα-RARα and RXRα-RXRα, only the results of rep1 (Table S1) are presented here for clarity but the results for the rest of the replicas are almost identical. In the case of RXRα-RXRα, the different color pairs correspond to homodimer with the wild-type (red) variant in both monomers, RXRα^{S427F} variant in one of the monomers (blue), and RXRα^{S427F} variant in both of the monomers (green). The position of the maximum pick of the different distributions is given in the tables below the graphs. (B) Graphical representation of the measured angled. The higher the value of the angle the more straight the underlying helix is. The position of the kink (Leu422) of H10 is shown in spheres.

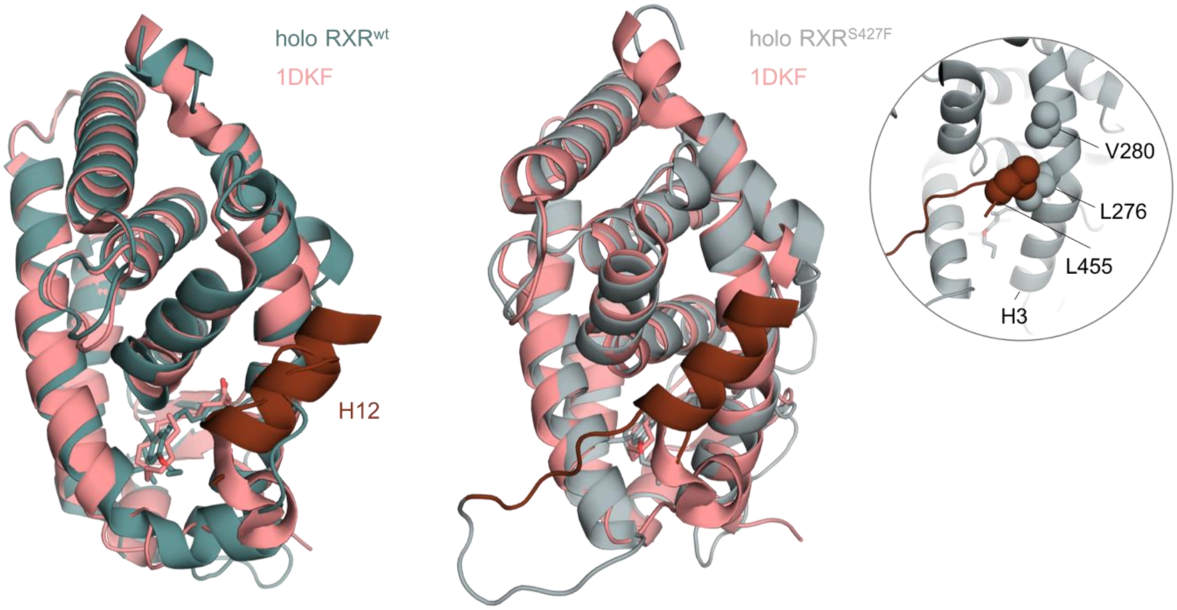

Figure S7. Comparison of the most frequently sampled conformations of the holo RXRα^{WT}/LG100754 (dark green) and RXRα^{S427F}/LG100754 (grey) from the RXRα-RARα simulations. Comparison of the most frequently sampled conformations of the holo RXRα^{WT}/LG100754 (dark green) and RXRα^{S427F}/LG100754 (grey) from the RXRα-RARα simulations, with the crystal structure of the RXRα^{WT} bound to the agonist, oleic acid (pink, PDB ID 1DKF). H12 (brown) in the crystal structure and RXRα^{WT} assume an auto-inhibitory position, where H12 binds to the co-activator's binding site. The simulations started from the antagonist conformation where H12 points to the solvent (PDB ID 3A9E, Figure S4).

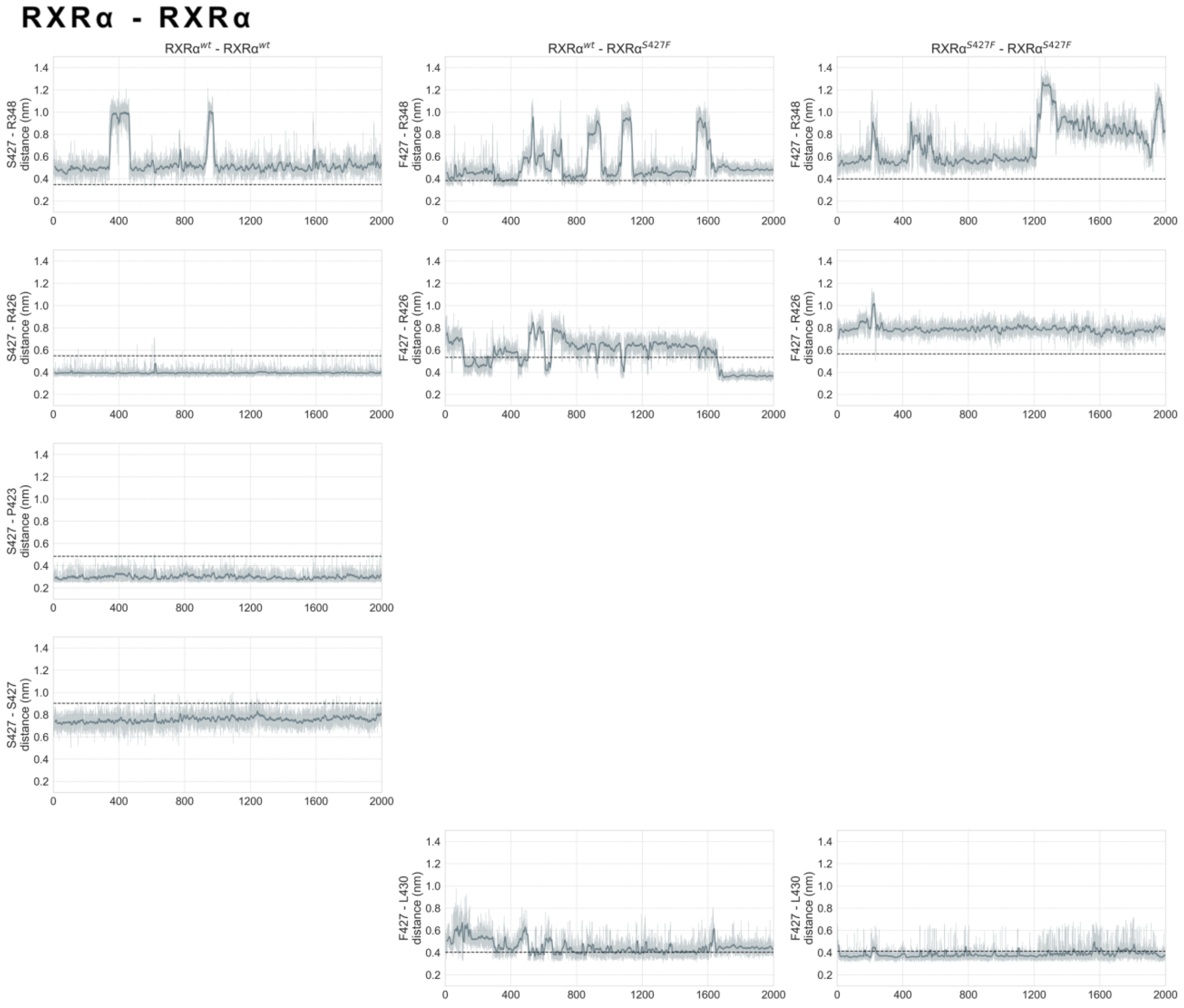

Figure S8. Time series of the Ser427 and Phe427 interactions in the RXRα homodimer. Timeseries of the interactions that Ser427$_{(OG)}$ and Phe427$_{(\text{center of mass of benzyl ring})}$ engage in over the course of the simulations of the apo RXRα^{WT}-RXRα^{WT}, apo RXRα^{WT}-RXRα^{S427F} and apo RXRα^{S427F}-RXRα^{S427F}. Residues Arg348$_{(CZ)}$ and Pro423$_{(O)}$ belong to the same RXRα monomer, while residues Ser427$_{(OG)}$, Leu430$_{(CD1)}$ and Arg426$_{(CZ)}$ belong to a different RXRα monomer. Thick traces represent moving averages, whereas thin traces represent original, unsmoothed values. Dashed horizontal lines indicate distance values in the corresponding crystal structures (PDB ID 1MVC).

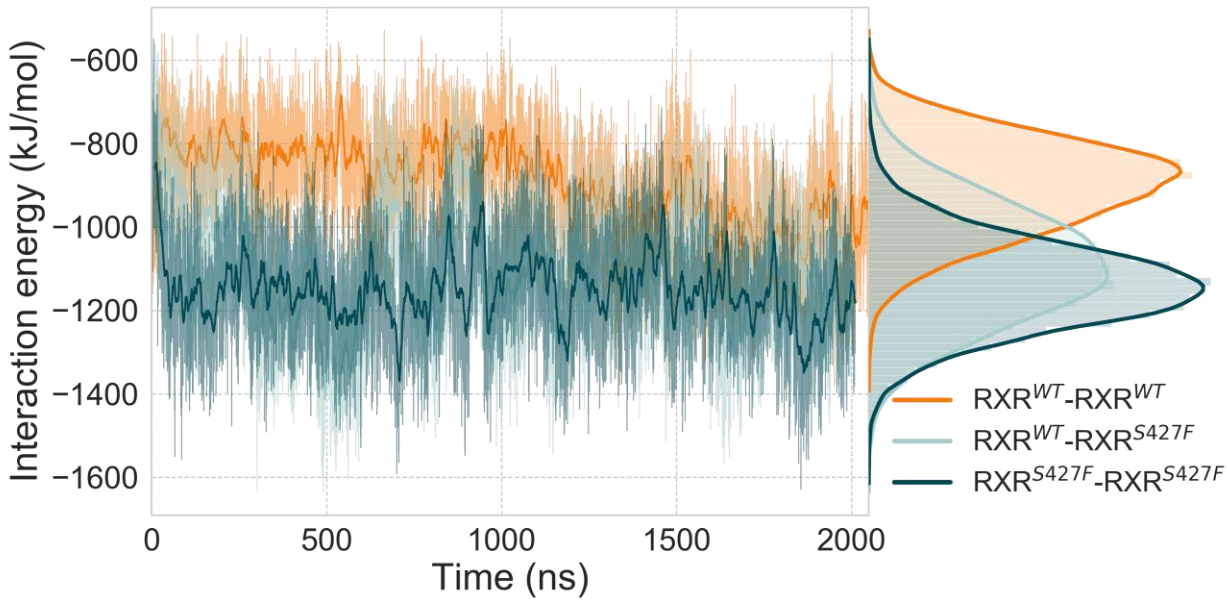

Figure S9. **Interaction energy between RXRα monomers over the course of the RXRα-RXRα simulations.** The reported energy corresponds to the sum of the intermolecular Coulombic and Lennard Jones potentials.

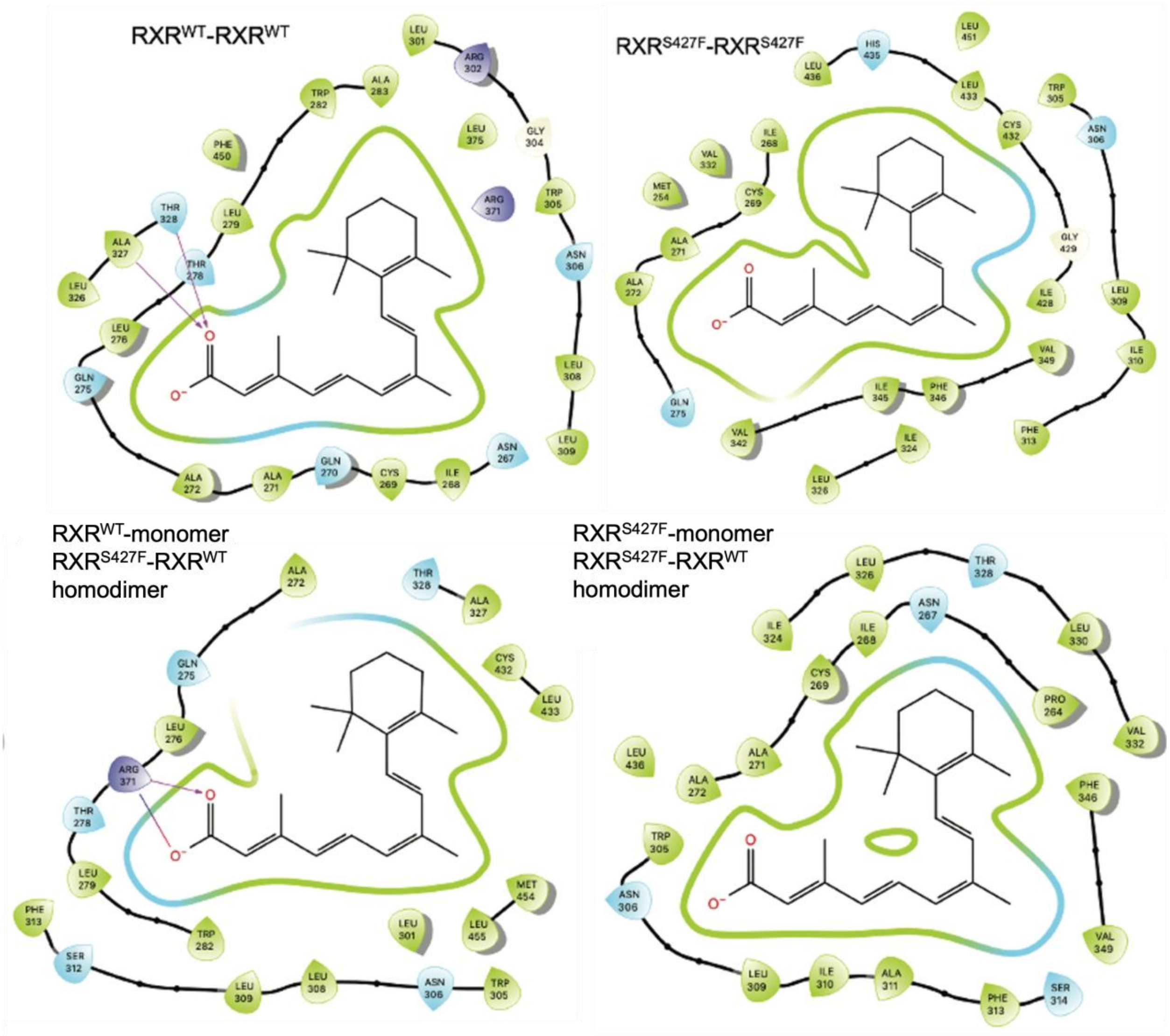

Figure S10. Ligand Interaction diagram of 9-cis RA bound to each RXRα monomer of the RXRα^{WT}-RXRα^{WT}, RXRα^{S427F}-RXRα^{S427F} and RXRα^{S427F}-RXRα^{WT} homodimers.

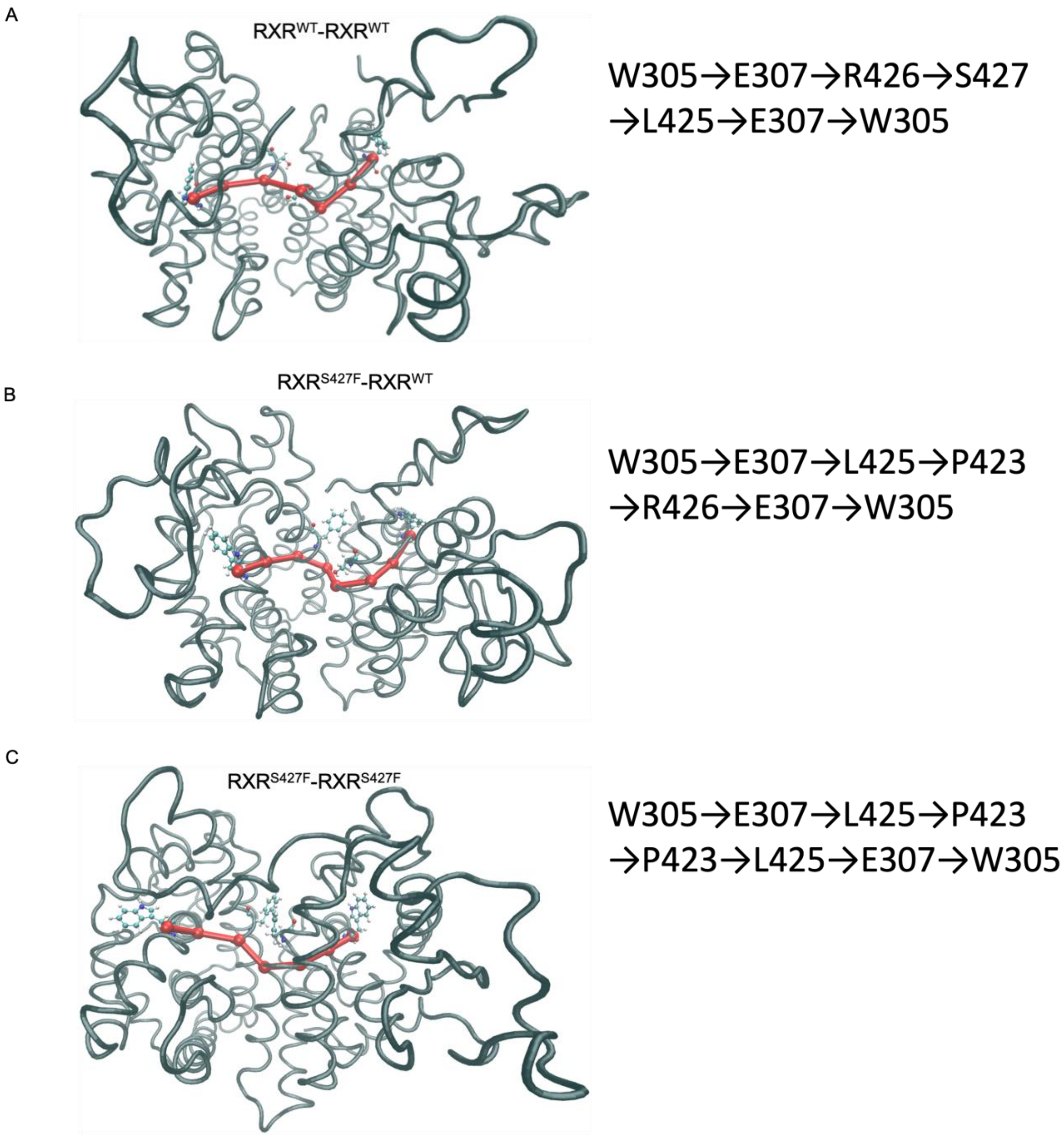

Figure S11. Suboptimal path of Trp305-Trp305 resulted from the dynamical network analysis performed on RXRα^{WT}-RXRα^{WT} (A), RXRα^{S427F}-RXRα^{S427F} (B) and RXRα^{S427F}-RXRα^{WT} (C) homodimers.

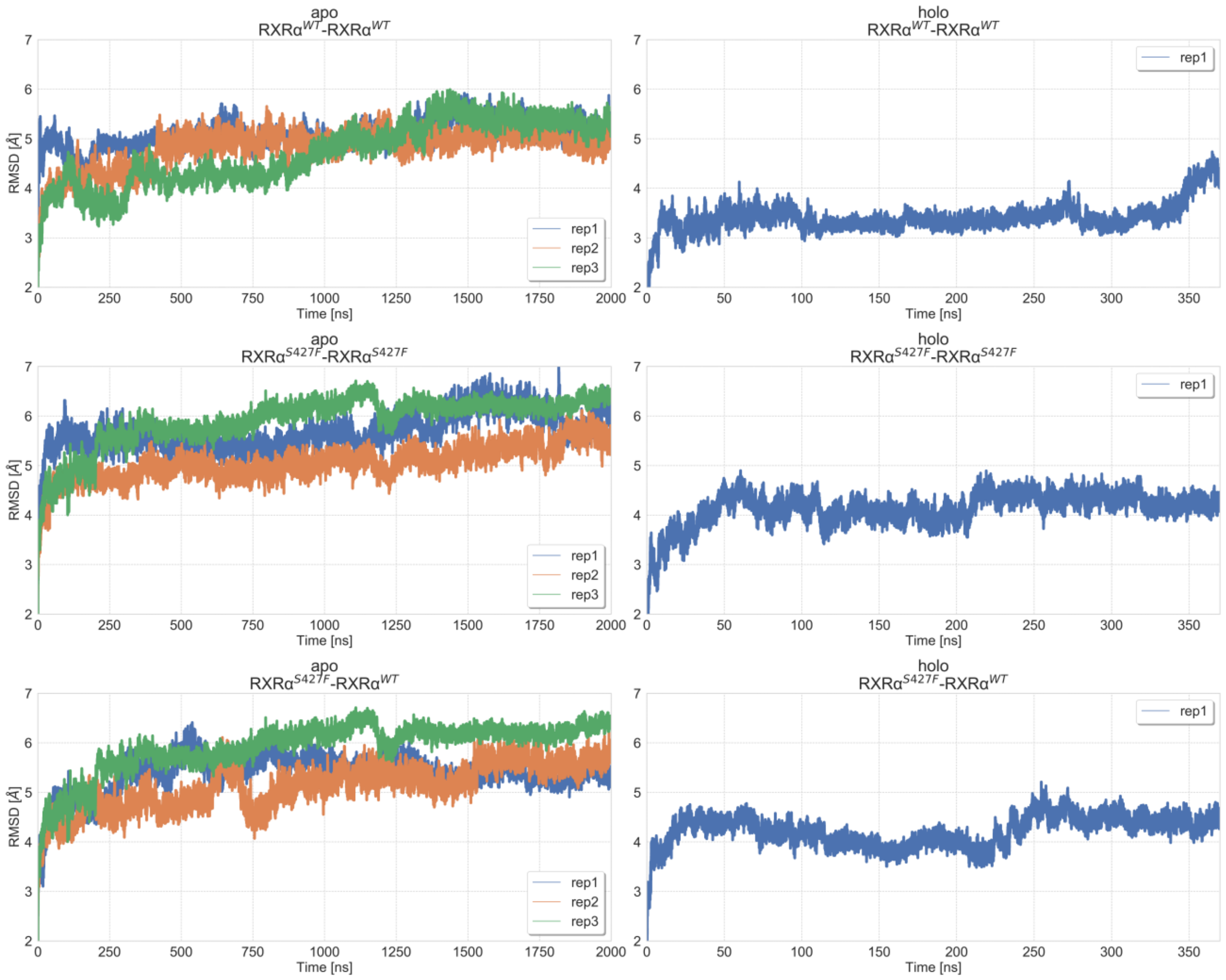

Figure S12. RMSD plot as a function of time of the RXR homodimer over the course of the RXRα-RXRα simulations.

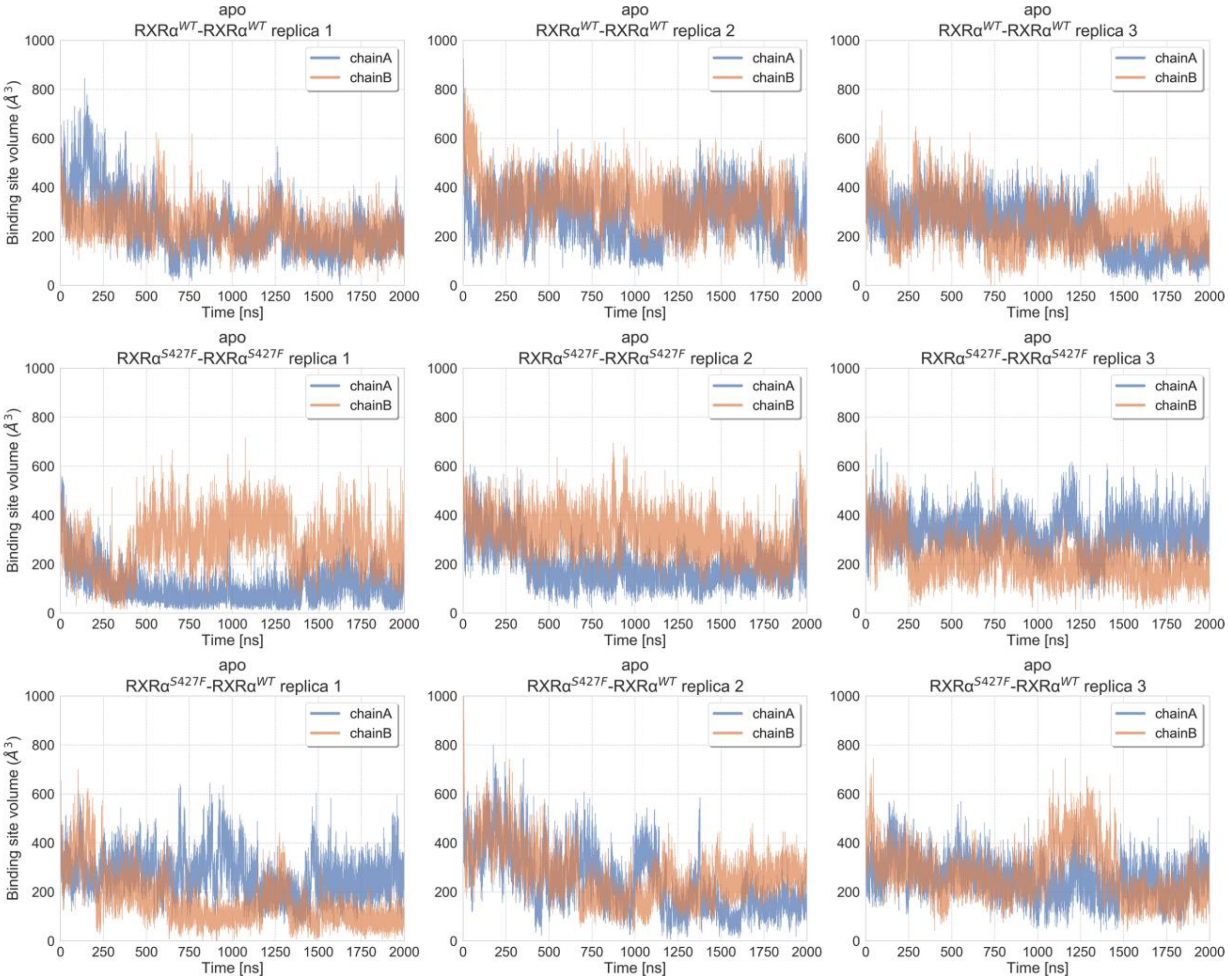

Figure S13. Plot of the volume of the binding site for each RXRα monomer as a function of time over the course of the RXRα-RXRα simulations. The left RXRα monomer (chain A) is colored with blue and right RXRα monomer is colored in orange.

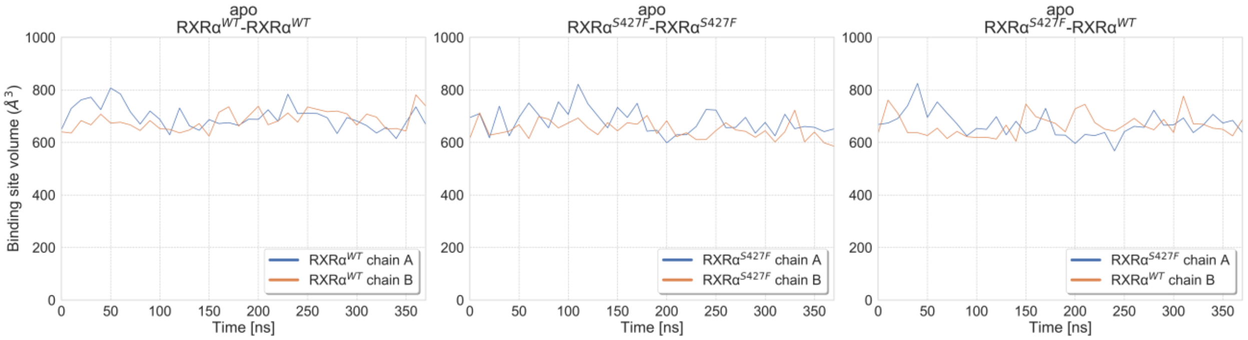

Figure S14. **Plot of the volume of the binding site for each RXRα monomer as a function of time over the course of the RXRα-RXRα simulations.** The RXRα monomer that belongs to chain A is colored with blue while the RXRα monomer that belongs to chain B is colored in orange.

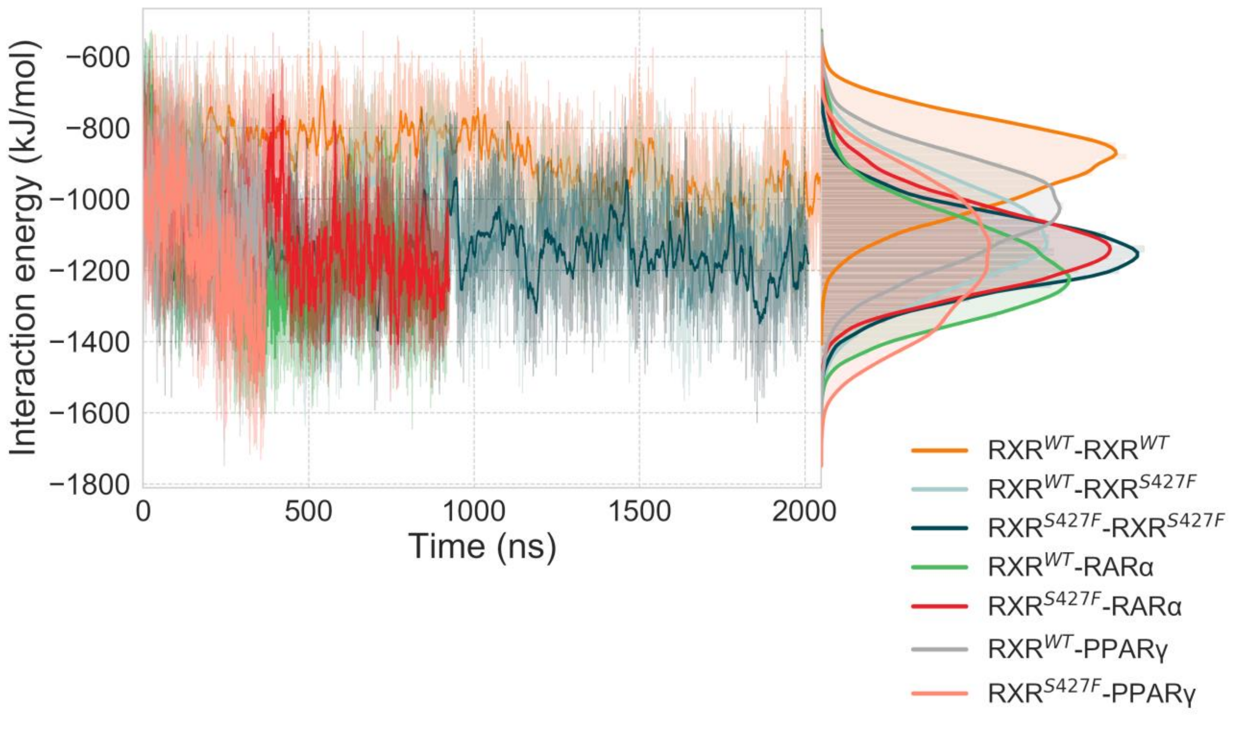

Figure S15. **Interaction energy between RXRα monomers and its homodimeric and heterodimeric partners over the course of the simulations.** The reported energy corresponds to the sum of the intermolecular Coulombic and Lennard Jones potentials.

RXRα - PPARγ

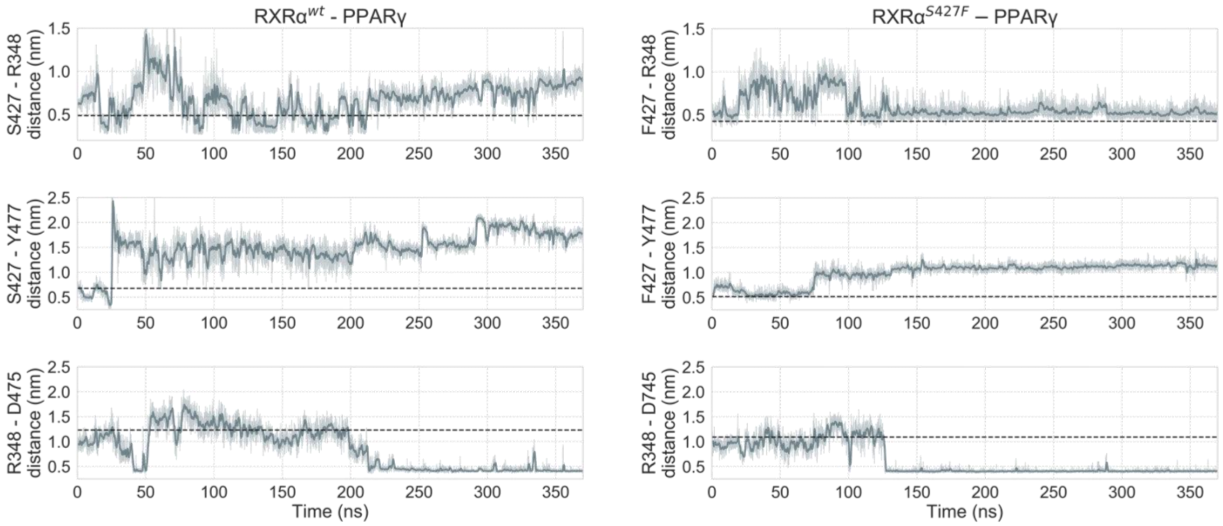

Figure S16. Timeseries of the interactions that Ser427 and Phe427 engage in over the course of the simulations of the apo RXRα^{WT}-PPRAγ, and apo RXRα^{S427F}-PPARγ. Timeseries of the interactions that Ser427(OG) and Phe427($_{\text{centre of mass of benzyl ring}}$) engage in over the course of the simulations of the apo RXRα^{WT}-PPRAγ, and apo RXRα^{S427F}-PPARγ. Residues Arg348$_{(CZ)}$ belong to the RXRα monomer, while residues Tyr477$_{(\text{centre of mass of benzyl ring})}$, and Leu430$_{(CD1)}$ belong PPARγ. Thick traces represent moving averages, whereas thin traces represent original, unsmoothed values. Dashed horizontal lines indicate distance values in the corresponding crystal structures of RXRα^{WT}-PPRAγ (PDB ID 1FM6) and RXRα^{S427F}-PPRAγ (PDB ID 5JI0).

```
                           Helices 10/11              Helix 12
                     hhhhhhhhhhhhhhhhhhhhhhhhhh-hhhh    hhhhhhh
P19793|RXRA_HS     PGRFAKLLLRLPALRSIGLKCLEHLFFF-KLIGDTPIDTFLMEMLEAPHQMT--------
P10276|RARA_HS     PHMFPKMLMKITDLRSISAKGAERVITL-KMEIPGSMPPLIQEMLENSEGLDTLSGQPGG
P37231|PPARG_HS    SQLFAKLLQKMTDLRQIVTEHVQLLQVIKKTETDMSLHPLLQEIYKDLY----------
                                  S427                             Y477
```

Figure S17. Sequence alignment of helices 10 to 12 of RXRα and its heterodimer partners RARα and PPARγ. Sequence alignment of helices 10 to 12 of RXRα and its heterodimer partners RARα and PPARγ (UniProt code | Receptor_Organism (HS: homo sapiens) Sequence). Residues that belong to a helix are annotated with a "h" on top of them. Of the three RXRα dimer partners, only PPARγ has an aromatic Tyr residue at the C-terminus that is able to form a π-stacking interaction with S427F mutation of RXRα.

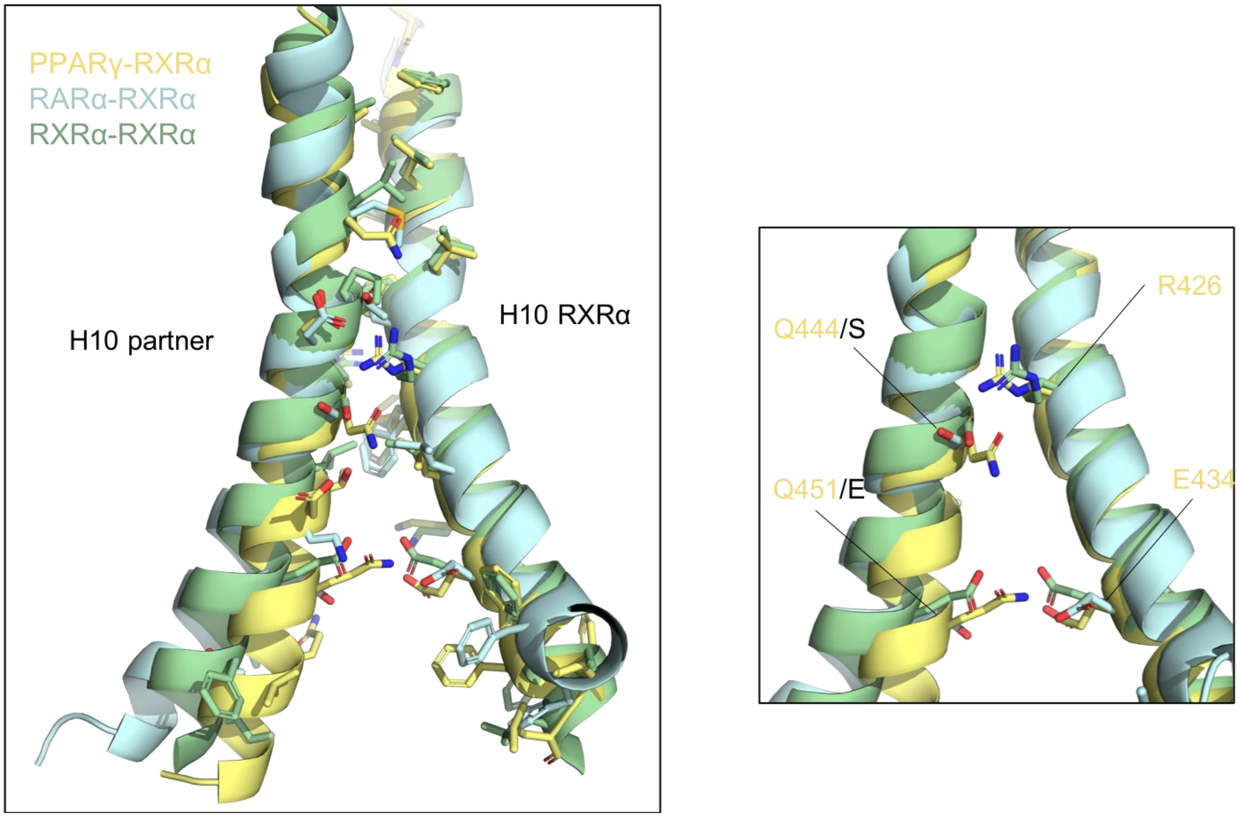

Figure S18. Superposition of H10 of RXRα, RARα, and PPARγ. The Q444PPARγ-R426RXRα and Q451PPARγ-E434RXRα interactions found on the interface of the RXRα-PPARγ heterodimer (close up) are expected to keep H10 of the two monomers closer together with respect to the RXRα- RARα and RXRα-RXRα.

References

(1) Egea, P. F.; Mitschler, A.; Moras, D. Molecular Recognition of Agonist Ligands by RXRs. *Mol. Endocrinol.* **2002**, *16* (5), 987–997. https://doi.org/10.1210/mend.16.5.0823.

(2) Jacobson, M. P.; Pincus, D. L.; Rapp, C. S.; Day, T. J. F.; Honig, B.; Shaw, D. E.; Friesner, R. A. A Hierarchical Approach to All-Atom Protein Loop Prediction. *Proteins Struct. Funct. Genet.* **2004**, *55* (2), 351–367. https://doi.org/10.1002/prot.10613.

(3) Jacobson, M. P.; Friesner, R. A.; Xiang, Z.; Honig, B. On the Role of the Crystal Environment in Determining Protein Side-Chain Conformations. *J. Mol. Biol.* **2002**, *320* (3), 597–608. https://doi.org/10.1016/S0022-2836(02)00470-9.

(4) Dolinsky, T. J.; Nielsen, J. E.; McCammon, J. A.; Baker, N. A. PDB2PQR: An Automated Pipeline for the Setup of Poisson-Boltzmann Electrostatics Calculations. *Nucleic Acids Res.* **2004**, *32* (WEB SERVER ISS.), 665–667. https://doi.org/10.1093/nar/gkh381.

(5) Sato, Y.; Ramalanjaona, N.; Huet, T.; Potier, N.; Osz, J.; Antony, P.; Peluso-Iltis, C.; Poussin-Courmontagne, P.; Ennifar, E.; Mély, Y.; et al. The "Phantom Effect" of the Rexinoid LG100754: Structural and Functional Insights. *PLoS One* **2010**, *5* (11). https://doi.org/10.1371/journal.pone.0015119.

(6) Altschul, S. F.; Wootton, J. C.; Gertz, E. M.; Agarwala, R.; Morgulis, A.; Schäffer, A. A.; Yu, Y. K. Protein Database Searches Using Compositionally Adjusted Substitution Matrices. *FEBS J.* **2005**, *272* (20), 5101–5109. https://doi.org/10.1111/j.1742-4658.2005.04945.x.

(7) Gampe, R. T.; Montana, V. G.; Lambert, M. H.; Miller, A. B.; Bledsoe, R. K.; Milburn, M. V.; Kliewer, S. A.; Willson, T. M.; Xu, H. E. Asymmetry in the PPARγ/RXRα Crystal Structure Reveals the Molecular Basis of Heterodimerization among Nuclear Receptors. *Mol. Cell* **2000**, *5* (3), 545–555. https://doi.org/10.1016/S1097-2765(00)80448-7.

(8) Halstead, A. M.; Kapadia, C. D.; Bolzenius, J.; Chu, C. E.; Schriefer, A.; Wartman, L. D.; Bowman, G. R.; Arora, V. K. Bladder-Cancer-Associated Mutations in RXRA Activate Peroxisome Proliferator-Activated Receptors to Drive Urothelial Proliferation. *Elife* **2017**, *6*, 1–27. https://doi.org/10.7554/eLife.30862.

(9) Harder, E.; Damm, W.; Maple, J.; Wu, C.; Reboul, M.; Xiang, J. Y.; Wang, L.; Lupyan,

D.; Dahlgren, M. K.; Knight, J. L.; et al. OPLS3: A Force Field Providing Broad Coverage of Drug-like Small Molecules and Proteins. *J. Chem. Theory Comput.* **2016**, *12* (1), 281–296. https://doi.org/10.1021/acs.jctc.5b00864.

(10) Price, D. J.; Brooks, C. L. A Modified TIP3P Water Potential for Simulation with Ewald Summation. *J. Chem. Phys.* **2004**, *121* (20), 10096–10103. https://doi.org/10.1063/1.1808117.

(11) Vanquelef, E.; Simon, S.; Marquant, G.; Garcia, E.; Klimerak, G.; Delepine, J. C.; Cieplak, P.; Dupradeau, F. Y. R.E.D. Server: A Web Service for Deriving RESP and ESP Charges and Building Force Field Libraries for New Molecules and Molecular Fragments. *Nucleic Acids Res.* **2011**, *39* (SUPPL. 2), 511–517. https://doi.org/10.1093/nar/gkr288.

(12) Dupradeau, F. Y.; Pigache, A.; Zaffran, T.; Savineau, C.; Lelong, R.; Grivel, N.; Lelong, D.; Rosanski, W.; Cieplak, P. The R.E.D. Tools: Advances in RESP and ESP Charge Derivation and Force Field Library Building. *Phys. Chem. Chem. Phys.* **2010**, *12* (28), 7821–7839. https://doi.org/10.1039/c0cp00111b.

(13) Bayly, C. I.; Cieplak, P.; Cornell, W. D.; Kollman, P. A. A Well-Behaved Electrostatic Potential Based Method Using Charge Restraints for Deriving Atomic Charges: The RESP Model. *J. Phys. Chem.* **1993**, *97* (40), 10269–10280. https://doi.org/10.1021/j100142a004.

(14) Wang, J.; Wolf, R. M.; Caldwell, J. W.; Kollman, P. A.; Case, D. A. Development and Testing of a General Amber Force Field. *J. Comput. Chem.* **2004**, *25* (9), 1157–1174. https://doi.org/10.1002/jcc.20035.

(15) Sousa Da Silva, A. W.; Vranken, W. F. ACPYPE - AnteChamber PYthon Parser InterfacE. *BMC Res. Notes* **2012**, *5*, 1–8. https://doi.org/10.1186/1756-0500-5-367.

(16) Abraham, M. J.; Murtola, T.; Schulz, R.; Páll, S.; Smith, J. C.; Hess, B.; Lindah, E. Gromacs: High Performance Molecular Simulations through Multi-Level Parallelism from Laptops to Supercomputers. *SoftwareX* **2015**, *1–2*, 19–25. https://doi.org/10.1016/j.softx.2015.06.001.

(17) Lindorff-Larsen, K.; Piana, S.; Palmo, K.; Maragakis, P.; Klepeis, J. L.; Dror, R. O.; Shaw, D. E. Improved Side-Chain Torsion Potentials for the Amber Ff99SB Protein Force Field.

Proteins Struct. Funct. Bioinforma. **2010**, *78* (8), 1950–1958. https://doi.org/10.1002/prot.22711.

(18) Duan, Y.; Wu, C.; Chowdhury, S.; Lee, M. C.; Xiong, G.; Zhang, W.; Yang, R.; Cieplak, P.; Luo, R.; Lee, T.; et al. A Point-Charge Force Field for Molecular Mechanics Simulations of Proteins Based on Condensed-Phase Quantum Mechanical Calculations. *J. Comput. Chem.* **2003**, *24* (16), 1999–2012. https://doi.org/10.1002/jcc.10349.

(19) Essmann, U.; Perera, L.; Berkowitz, M. L.; Darden, T.; Lee, H.; Pedersen, L. G. A Smooth Particle Mesh Ewald Method. *J. Chem. Phys.* **1995**, *103* (19), 8577–8593. https://doi.org/10.1063/1.470117.

(20) Parrinello, M.; Rahman, A. Polymorphic Transitions in Single Crystals: A New Molecular Dynamics Method. *J. Appl. Phys.* **1981**, *52* (12), 7182–7190. https://doi.org/10.1063/1.328693.

(21) Nosé, S. A Molecular Dynamics Method for Simulations in the Canonical Ensemble. *Mol. Phys.* **1984**, *52* (2), 255–268. https://doi.org/10.1080/00268978400101201.

(22) Daura, X.; Van Gunsteren, W. F.; Mark, A. E.; Gademann, K.; Jaun, B.; Seebach, D. Peptide Folding: When Simulation Meets Experiment. *Angew. Chemie - Int. Ed.* **1999**, *38* (1–2), 236–240. https://doi.org/10.1002/(SICI)1521-3773(19990115)38:1/2<236::AID-ANIE236>3.0.CO;2-M.

(23) Wilman, H. R.; Shi, J.; Deane, C. M. Helix Kinks Are Equally Prevalent in Soluble and Membrane Proteins. *Proteins Struct. Funct. Bioinforma.* **2014**, *82* (9), 1960–1970. https://doi.org/10.1002/prot.24550.

(24) Zhang, H.; Chen, L.; Chen, J.; Jiang, H.; Shen, X. Structural Basis for Retinoic X Receptor Repression on the Tetramer. *J. Biol. Chem.* **2011**, *286* (28), 24593–24598. https://doi.org/10.1074/jbc.M111.245498.

(25) Bourguet, W.; Vivat, V.; Wurtz, J. M.; Chambon, P.; Gronemeyer, H.; Moras, D. Crystal Structure of a Heterodimeric Complex of RAR and RXR Ligand-Binding Domains. *Mol. Cell* **2000**, *5* (2), 289–298. https://doi.org/10.1016/S1097-2765(00)80424-4.
